# Selectivity Screening and Structure–Cytotoxic Activity Observations of Selected Oleanolic Acid (OA)-Type Saponins from the Amaranthaceae Family on a Wiade Panel of Human Cancer Cell Lines

**DOI:** 10.3390/molecules29163794

**Published:** 2024-08-10

**Authors:** Karolina Grabowska, Agnieszka Galanty, Łukasz Pecio, Anna Stojakowska, Janusz Malarz, Paweł Żmudzki, Paweł Zagrodzki, Irma Podolak

**Affiliations:** 1 Department of Pharmacognosy, Jagiellonian University Medical College, 9 Medyczna Str., 30-688 Cracow, Poland; karolina1.grabowska@uj.edu.pl (K.G.); irma.podolak@uj.edu.pl (I.P.); 2 Department of Biochemistry and Crop Quality, Institute of Soil Science and Plant Cultivation–State Research Institute, ul. Czartoryskich 8, 24-100 Puławy, Poland; lukasz.pecio@gmail.com; 3 Department of Chemistry of Natural Products, Medical University of Lublin, 20-093 Lublin, Poland; 4 Maj Institute of Pharmacology, Polish Academy of Sciences, Smętna Street 12, 31-343 Kraków, Poland; stoja@if-pan.krakow.pl (A.S.); malarzj@if-pan.krakow.pl (J.M.); 5 Department of Medicinal Chemistry, Jagiellonian University Medical College, 9 Medyczna Str., 30-688 Cracow, Poland; pawel.zmudzki@uj.edu.pl; 6 Center for the Development of Therapies for Civilization and Age-Related Diseases, Jagiellonian University Medical College, Skawińska 8, 31-066 Krakow, Poland; 7 Department of Food Chemistry and Nutrition, Jagiellonian University Medical College, 9 Medyczna, 30-688 Kraków, Poland; pawel.zagrodzki@uj.edu.pl

**Keywords:** *Chenopodium strictum*, calenduloside E, chikusetsusaponin IVa, momordin Ic, cytotoxic, structure–activity, saponins

## Abstract

Plants from the Amaranthaceae family are a source of oleanolic acid (OA)-type saponins with cytotoxic activity. Two known OA-type saponins, calenduloside E and chikusetsusaponin IVa, were isolated from the roots of *Chenopodium strictum* Roth. Their structures were confirmed using MS and NMR techniques. This constitutes the inaugural report of the saponins in *Ch. strictum*. Both the isolated saponins and structurally similar compounds, momordin Ic and OA, were compared for their cytotoxicity against various cancer and normal cell lines (including skin, breast, thyroid, gastrointestinal, and prostate panels). Their effects were dose- and time-dependent, varying with the specific cell line and compound structure. A chemometric approach demonstrated the effects of the compounds on the cell lines. The study discusses the structure–activity observations. The key structural elements for potent cytotoxic activity included the free carboxyl group 28COOH in the sapogenin structure (OA) and the presence of a sugar moiety. The monodesmosides with glucuronic acid (GlcA) at the C3 position of OA were generally more cytotoxic than bidesmosides or OA alone. The addition of xylose in the sugar chain modified the activity towards the cancer cells depending on the specific cell line. OA-type saponins with GlcA (particularly calenduloside E and momordin Ic) represent a promising avenue for further investigation as potential anticancer agents.

## 1. Introduction

Triterpene saponins represent a vast group of plant metabolites with a multitude of biological activities. They can be classified into numerous groups and subgroups based on their aglycone type and the structure of the sugar moiety. Given the structural diversity of saponins, they are the subject of intensive investigation with regard to their biological activities, which include anti-inflammatory, adaptogenic, antimicrobial, antidiabetic, antiviral, and hepatoprotective effects [1,2,3,4,5]. One of the most promising areas of research in the field of triterpene saponin bioactivity is the investigation of their potential to induce cytotoxicity in cancer cell lines in vitro, as well as their anti-tumor effects [2,6]. Triterpene saponins are present in a wide range of plant families, including the Amaranthaceae family [7]. These are primarily oleanolic acid-type monodesmosidic or bidesmosidic derivatives, which have received attention due to their anticancer activity in both in vitro and in vivo testing [6,7,8]. 

In the pursuit of novel, sustainable sources of these bioactive saponins, our research has focused on an as yet uninvestigated species: *Chenopodium strictum* Roth (syn. C*henopodium betaceum* Andrz.) [9]. *Chenopodium strictum* Roth is an annual plant belonging to the genus *Chenopodium*, which is widely distributed in North America, Europe, and Asia. It is commonly known as late-flowering goosefoot or striped goosefoot. The only published reports on the phytochemical composition of this plant species demonstrated the presence of flavonoids and proteins [10,11,12]. However, no previous data refer to saponins.

This study reports the first isolation of oleanolic acid (OA)-type saponins from *Ch. strictum*: calenduloside E (CE) and chikusetsusaponin IVa (ChIVa). Both compounds possess a glucuronic acid moiety at the C-3 position of the sapogenin. However, ChIVa differs from CE in that it contains an additional ester-linked glucose residue via the C-28 carboxyl group of OA. It may be significant to examine the impact of these compounds on cancer cells given that some authors have proposed that they may serve as metabolites of the more complex OA saponins and contribute to the observed effects in vivo [8]. Moreover, the distinctions between these two saponins are noteworthy in the context of the structure–activity relationship (SAR). 

Even though both compounds have previously been shown to exert cytotoxic effects in several cancer cell lines (calenduloside E against HeLA, MCF-7, A549, Lovo, LN229, WiDr, B16F10, and HepG2 cells [8,13,14,15,16], whereas chikusetsusaponin IVa against HL-60, HCT-116, A2780, A2780, HEY, SK -Hep-1, HT-29, PC-3, LNCaP, Du145, HEC1B, and A549 cells [8,14,17,18,19,20,21]), only two comparative analyses on the anticancer potential of these saponins have been carried out so far. One study investigated the effect of the compounds on WiDr and MCF-7 cancer cells [14], while another examined the inhibitory effect on melanoma growth in 16F10 tumor-bearing mice [8]. It is noteworthy that the cytotoxic activity of isolated saponins has not yet been compared with other compounds with a related structure, such as oleanolic acid, or saponins with an analogous structure and documented cytotoxic activity, such as momordin Ic (MIc).

Momordin Ic is the predominant saponin present in the seeds of *Bassia scoparia* (*Kochiae fructus*), a plant belonging to the Amaranthaceae family [22]. MIc differs from calenduloside E solely in the presence of an additional xylose residue situated at the C-3′ position of the GlcA. The activity of this compound was primarily evaluated in models of prostate and gastrointestinal cancer. A number of published studies indicate that MIc may possess anticancer properties and exerts its effects through a range of mechanisms, including the PI3K/Akt-mediated pathways or MAPK-dependent PPARγ activation [23,24,25]. Recent studies have demonstrated that MIc functions as an inhibitor of an SUMO protease isoform, SENP1. The overexpression of SENP1 has been observed in certain types of cancer, including prostate, lung, blood, and colon cancers [26]. 

A substantial body of evidence suggests that the structural characteristics of a compound can influence its cytotoxic potency against cancer cells [6,27,28]. Nevertheless, the research on OA-type sapogenins containing GlcA in the sugar moiety remains scarce. Furthermore, the impact of the structural characteristics on the activity of these compounds towards normal cells is rarely explored. The primary objective of this study, in addition to the isolation and structural elucidation of saponins from *Ch. strictum* Roth, was to examine their cytotoxicity and selectivity in a wide range of human cell lines (cancerous and normal). Furthermore, the study sought to examine the relationship between the cytotoxic activity and compound structure. In addition to the isolated saponins, namely calenduloside E and chikusetsusaponin IVa, momordin Ic was also subjected to analysis, as was oleanolic acid, which represents the aglycone of these saponins. The study therefore encompasses a series of analogues with the potential to exert cytotoxic effects. To the best of our knowledge, comparative studies on the cytotoxic effects and selectivity of these compounds have not been conducted previously.

## 2. Results and Discussion

### 2.1. Isolation and Structure Elucidation

In the initial phase of the study, the roots, leaves, stems, and fruits of *Ch. strictum* Roth were extracted in a sequential manner using solvents of increasing polarity, commencing with chloroform and subsequently methanol. The preliminary TLC analysis of the methanolic extracts indicated the presence of saponins in the roots of *Ch. strictum* (Figure S1). Accordingly, this morphological portion of the plant was selected for further analysis. To isolate the saponins, a methanolic root extract was prepared. Subsequently, the extract was suspended in water and partitioned with *n*-BuOH. A liquid–liquid partitioning process yielded a butanolic extract that was rich in saponins. This extract was then subjected to a series of purification steps. In order to obtain compounds with a purity level exceeding 95% (as confirmed by UPLC-PDA), it was necessary to employ a range of chromatographic techniques, including column chromatography (CC), medium-pressure liquid chromatography (MPLC), and preparative high-pressure liquid chromatography (prep-HPLC), in addition to utilizing diverse stationary phases (NP and RP) and mobile phases (for further details, please refer to the Section 3, Materials and Methods ). Consequently, two compounds (designated as **1** and **2**) were obtained in the form of a white powder. The structure of these compounds was determined through the analysis of the spectral data obtained from 1D and 2D NMR, as well as ESI-MS and the fragmentation pathways of the compounds (for details, please see the Supplementary Materials).

The data obtained, when compared with the existing literature [29,30,31,32], indicated that compound **1** is identical with oleanolic acid-3-*O*-*β*-d-glucuronopyranoside (calendulo-side E), and compound **2** is 3-*O*-*β*-d-glucuronopyranosyl oleanolic acid 28-*O*-*β*-d-glucopyranosyl ester, also known as chikusetsusaponin IVa. 

The presence of calenduloside E and chikusetsusaponin IVa has already been documented in other plants belonging to the Amaranthaceae family. The following species have been identified as containing calenduloside E: *Achyranthes bidentata* [33], *Bassia muricata*, *Chenopodium album* L., *Chenopodium quinoa*, *Salicornia bigelovii*, *Salicornia europaea*, *Atriplex nummularia* [7], and *Atriplex sagitatta* [32]. Chikusetsusaponin IV has been isolated from the following species: *Achyranthes faureri*, *Alternanthera philoxeroides*, *Bassia muricata*, *Chenopodium album*, *Salicornia bigelovii* [7], and *Atriplex sagittata* [32]. Furthermore, its presence was identified by Q-TOF MS/MS in *Achyranthes bidentata* [34] and *Beta vulgaris* var. *vulgaris* (sugar beet) [29]. To the best of our knowledge, this is the first account of the presence of saponins in *Chenopodium strictum* Roth.

### 2.2. Cytotoxic Activity

**Figure 1:**
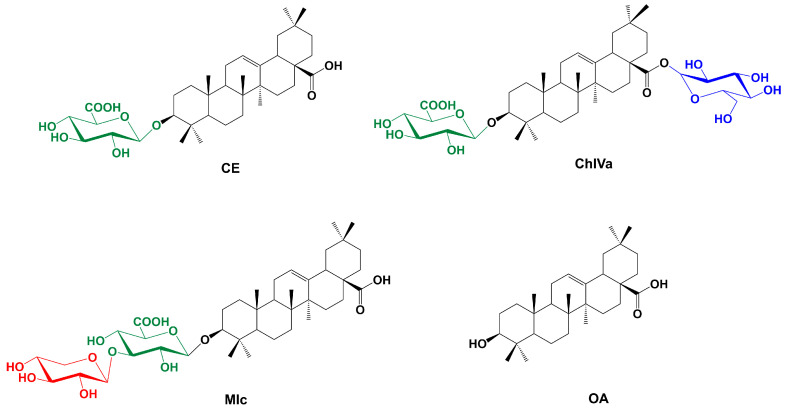
Structures of compounds: calenduloside E (CE), chikusetsusaponin IVa (ChIVa), momordin Ic (MIc), and oleanolic acid.

To compare the cytotoxic effects of a group of oleanolic acid analogues, in addition to the saponins isolated from *Ch. strictum* (CE and ChIVa), commercially available momordin Ic (oleanolic acid-3-*O*-[*β*-d-xylopyranosyl(1→3)*-β*-d-glucuronopyranoside]) and oleanolic acid, which is a sapogenin common to all three saponins analyzed, were also included in the study (Figure 1).

The cytotoxicity analysis, conducted using the MTT method, evaluated the impact of the four compounds on human cancer cells, organized into five panels: skin, gastrointestinal, prostate, thyroid, and breast. The cancer cell lines employed in the study exhibited varying degrees of malignancy and metastatic potential, thereby reflecting the phenotypic heterogeneity of the cancer cells and the complex nature of the tumors. Furthermore, in an effort to create an in vitro model that more closely approximates the natural conditions and to ascertain the selectivity of the compounds under examination, human normal cell lines (HaCaT; Nthy-ori 3-1, CCD841 CoN, MCF10A, and PNT2) were also included in the study. The study included both cell lines for which studies on the activity of OA analogues had been conducted, thus providing a basis for comparison, as well as cancer and non-cancer cells whose sensitivity to these compounds had not yet been assessed. Doxorubicin was employed as the reference cytotoxic substance. The data pertaining to the cytotoxic effects of the compounds, expressed as IC_50_ values, are presented in the tables (Table 1, Table 2, Table 3, Table 4 and Table 5). The study demonstrated that all the tested compounds affected the cancer cell viability in a manner that was dependent on both the dose and time. However, it was observed that the individual cell lines exhibited differing levels of sensitivity to a given compound.

#### 2.2.1. Prostate Panel

MIc was previously found to be an SENP1 inhibitor that suppresses PC3 cell proliferation both in vitro and in vivo [26]. Additionally, the work by Zhu et al. [21] showed that ChIVa induces apoptosis in human prostate cancer cells via reactive oxygen species (ROS) production. This therefore suggests that compounds with MIc-like structures may affect prostate cancer cells. More recently, the action of a series of titerpenoids, inclucing MIc, as potential SENP1 inhibitors was investigated by Zhang et al. [35]. It is noteworthy that the compounds used in this study, although all belonging to triterpenoids, had significantly different structures from MIc (except for OA). This approach identifies the most active structures but makes it difficult to determine the relationship between the structure and activity [35]. This prompted us to conduct a prostate panel study on MIc analogues, which include saponins with very similar structures (Figure 1). 

As expected, both of the compounds previously analyzed for their effects on prostate cancer cells, MIc and ChIVa, were active against PC3 and Du-145 cells (Table 1). However, the effect of ChIVa on the Du-145 line was four times weaker than that of MIc and comparable to the activity of OA. ChIVa also showed significantly weaker activity against the PC3 line compared to MIc. In turn, the CE activity against the PC3 cell line was comparable to MIc and only slightly lower against the Du-145 cell line. Surprisingly, the study showed that the compounds analyzed, including MIc, had a very weak effect on the androgen-sensitive human prostate adenocarcinoma cell line (LNCaP) (60–70% cell viability at a concentration 100 μg/mL). Moreover, it is worth noting that none of the compounds showed toxicity to normal prostate epithelial cells. Similar observations regarding the effects of MIc and ChIVa on another human prostate normal cell line (RWPE-2) have been reported previously [21,26]. It has been suggested that the strong activity of the MIc against prostate PC-3 cancer cells and its lower activity towards LNCaP and normal cells are closely related to the observed overexpression of SENP1 in cancer cells [26].

**Table 1:** IC_50_ values of CE, ChIVa, MIc, and OA on prostate cell lines.

IC_50_ [μg/mL]								
	Du-145		PC3		LNCaP		PNT2	
Comp.	24 h	48 h	24 h	48 h	24 h	48 h	24 h	48 h
CE	6.7	4.3	5.6	2.9	>100	>100	>100	>100
ChIVa	22.9	9.1	45.6	22.8	>100	>100	>100	>100
MIc	5.4	2.8	5.5	3.4	>100	>100	>100	>100
OA	23.4	12.2	>100	>100	>100	>100	>100	>100
DOX	3.2		>50		1.8		1.4	

Abbreviations: calenduloside E (CE); chikusetsusaponin IVa (ChIVa); momordin Ic (MIc); oleanolic acid (OA); reference drug (DOX).

#### 2.2.2. Breast Panel

The breast cancer model used included both hormone-positive (MCF-7) and hormone-negative (MDA-MB-231) cell lines. CE, MIc, and OA affect both breast cancer cell types in a dose-dependent manner, with the hormone-negative cell line being more sensitive than ER-positive cells to the compounds tested. It was shown that, after 24 h of incubation, CE and MIc showed weak activity against the ER-negative cell line (Table 2), with a significant increase in the activity after 48 h of incubation. As the MDA-MB-231 cell line is highly metastatic, the results obtained are very relevant for further studies. The current data are similar to the results of the previously published studies showing CE activity against MCF-7 cells at longer incubation times: 48 h (IC_50_ = 35.4 μg/mL) [13] (IC_50_ = 19.7 μM) [14], or even 72 h (IC_50_ = 13.4 μM) [15]. It is noteworthy that the cytotoxic effects of MIc and CE were selective as neither substance analyzed was toxic to a normal breast epithelial cell line (MCF10A), even at the highest concentration tested (100 μg/mL). Of the saponins, only ChIVa showed negligible activity (IC_50_ > 100 μg/mL) against both cancer lines in the breast panel, which is consistent with a previous study by Huang et al. [14].

**Table 2:** IC_50_ values of CE, ChIVa, MIc, and OA on breast cell lines.

IC_50_ [μg/mL]						
	MDA-MB-231		MCF7		MCF10A	
Comp.	24 h	48 h	24 h	48 h	24 h	48 h
CE	69.6	23.5	>100	39.5	>100	>100
ChIVa	>100	98.1	>100	>100	>100	>100
MIc	52.2	16.4	>100	29.2	>100	>100
OA	>100	38.5	>100	67.0	>100	>100
DOX						

Abbreviations: calenduloside E (CE); chikusetsusaponin IVa (ChIVa); momordin Ic (MIc); oleanolic acid (OA); reference drug (DOX).

#### 2.2.3. Thyroid Panel

**Figure 2:**
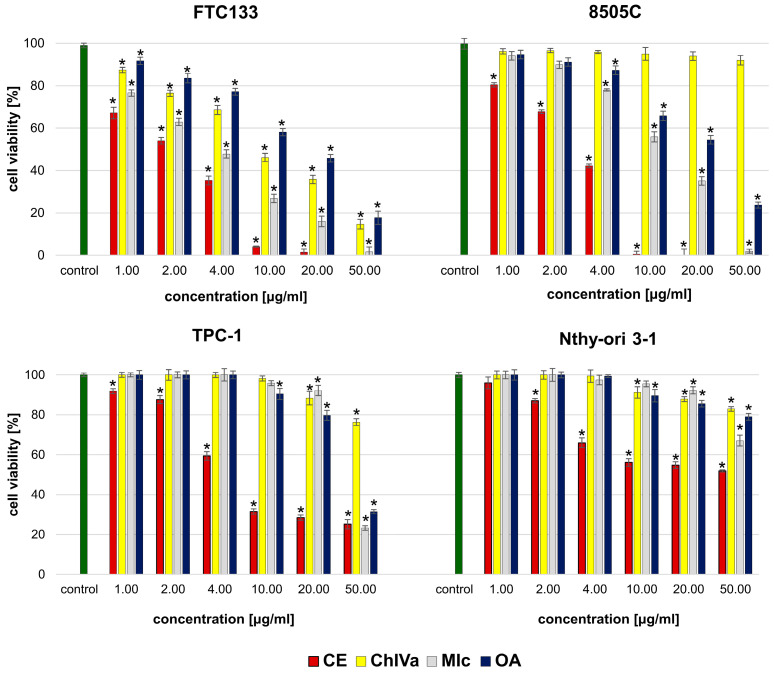
The cytotoxic effects of analyzed compounds: caleduloside E (CE), chikusetsusaponin IVa (ChIVa), momordin Ic (Mic), and oleanolic acid (OA) on cancerous (FTC133, 8505C, and TPC-1) and normal (Nthy-ori 3) thyroid cells. Values are presented as the mean ± SD (standard deviation). Results from the MTT viability assay after 24 h incubation with tested substances. Results are presented as the mean ± SD. The values significantly different from the control (untreated group) are indicated by * for *p* ˂ 0.05.

While OA activity has been studied on thyroid cells [36,37], to our knowledge, this is the first study on the effects of the tested saponins on both the normal and cancerous cells of the thyroid gland. We used three different human cancer cell lines representing different types of thyroid cancers: follicular thyroid carcinoma, lymph node metastasis (FTC133), human thyroid carcinoma, undifferentiated (8505C), and papillary thyroid carcinoma (TPC-1). Significant differences were found in the activity of the individual compounds against the lines analyzed, as illustrated in Figure 2. CE turned out to be the most active against all the cancer lines tested (Table 3). Already after 24 h of incubation, CE showed a strong cytotoxic effect against metastatic FTC133 cells, twice superior to the reference drug used. This saponin was also significantly more active than doxorubicin against the 8505C line, which represents a rare, aggressive anaplastic type of thyroid cancer. Although CE also had some effect on the viability of normal thyroid epithelial cells (Nthy-ori 3-1), its activity can be considered selective due to the high selectivity index (SI) value (Table 3). In contrast, its analogue, ChIVa, showed weak activity against most thyroid cancer cells, except for FTC133, to which it was moderately cytotoxic. Furthermore, it was found that MIc displayed potent activity against the FTC133 line, comparable to the reference drug, while demonstrating moderate activity against the other cancer cell lines. To sum up, the FTC133 line proved to be the most sensitive to the tested compounds, but their effectiveness, except for ChIVa, to 8505C anaplastic thyroid carcinoma should also be highlighted. This is extremely important as the latter cell line represents a thyroid cancer with a poor prognosis.

**Table 3:** IC_50_ values and selectivity index (SI) values of CE, ChIVa, MIc, and OA on thyroid cell lines.

IC_50_ [μg/mL] and (Selectivity Index (SI))								
	FTC133		8505C		TPC-1		Nthy-ori 3-1	
Comp.	24 h	48 h	24 h	48 h	24 h	48 h	24 h	48 h
CE	1.9 (38.6)	1.1 (28.1)	2.8 (26.9)	1.8 (16.5)	8.5 (9.0)	5.8 (5.1)	76.0	30.1
ChIVa	8.5 (11.76)	4.0 (25.1)	>100 (NA)	>100 (NA)	>100 (NA)	81.8 (1.2)	>100	>100
MIc	3.5 (24.85)	2.1 (30.7)	10.6 (8.2)	5.2 (12.2)	39.0 (2.2)	39.0 (1.6)	86.2	64.4
OA	13.5 (6.99)	4.9 (14.6)	19.5 (4.8)	11.3 (6.4)	36.6 (2.6)	30.0 (2.4)	94.4	71.6
DOX	4.0 (6.76)		>40		3.9 (7.0)		27.2	

Abbreviations: calenduloside E (CE); chikusetsusaponin IVa (ChIVa); momordin Ic (MIc); oleanolic acid (OA); reference drug (DOX).

#### 2.2.4. Gastrointestinal Panel

Colon cancer cells (Caco-2, DLD-1, HCT116), colorectal adenocarcinoma (HT29), and liver cancer cells (HepG2) were used to investigate the effects of the compounds on gastrointestinal tract cancers. A normal intestinal epithelial cell line (CCD841 CoN) was also included in the study to analyze the selectivity of the compounds. Previously published reports have indicated that MIc exerts cytotoxic activity against HepG2 cells by inducing apoptosis via the MAPK- and PI3K/Akt-mediated pathways [24], including PI3K- and MAPK-dependent PPARγ activation [25]. A study by Mi et al. [23] showed that the saponin induced apoptosis by suppressing the PI3K/Akt-dependent NF-κB pathways and also promoted autophagy by the ROS-mediated Erk signaling pathway. In our study, we confirmed the cytotoxic activity of MIc against the HepG2 line and revealed a slightly weaker activity of CE than MIc on HepG2, which can be described as moderate. 

In a study by Xianjun et al. [38], it was shown that MIc inhibits cell proliferation with G0/1 phase cell cycle arrest in the G0/1 phase and induces apoptosis in the HT-29 and HCT-116 cell lines. The mechanism of action of this saponin is related to the inhibition of the SENP1/c-Myc signalling pathway in intestinal cancer cells [38]. In our study, MIc turned out to be the most active against the HCT-116 cell line. In the HT-29 line, the MIc activity was comparable to CE, whereas the ChIVa activity was much lower than that of the other saponins. The observed effect of ChIVa on the HT-29 cells, after 48 h of incubation, is consistent with the data previously reported by Yen et al. [20].

**Figure 3:**
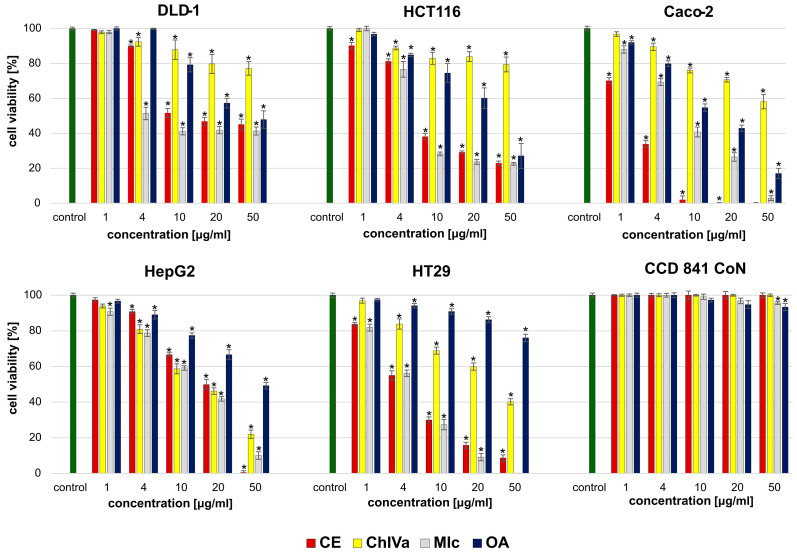
The cytotoxic effects of analyzed compounds: caleduloside E (CE), chikusetsusaponin IVa (ChIVa), momordin Ic (Mic), and oleanolic acid on cancerous (DLD1, HCT-116, Caco2, HepG2, and Ht29) and normal (CCD841CoN) colon cells. Results from the MTT viability assay after 24 h incubation. Results are presented as the mean ± SD. The values significantly different from the control (untreated group) are indicated by * for *p* ˂ 0.05.

It is worth noting that the different cell line types within the gastrointestinal panel differed significantly in their sensitivity to the analyzed substances, as shown in Figure 3. Overall, MIc showed the highest cytotoxic activity against most cancer cell lines. The exception, however, was the effect on Caco-2 cells, which turned out to be most sensitive to another saponin—CE. It is worth noting that the CE activity was significantly higher than that recorded for the reference drug (Table 4). Moreover, it is important to note that all the substances analyzed, used even at the highest dose of 100 μg/mL, caused the death of normal colon epithelial cells by only a maximum of 6%, indicating their high selectivity towards cancer cells and, consequently, safety. 

**Table 4:** IC_50_ values of CE, ChIVa, MIc, and OA on cell lines grouped in gastrointestinal panel.

IC_50_ [μg/mL]												
	Caco-2		HT29		HepG2		DLD-1		HCT116		CCD 841 CoN	
Comp.	24 h	48 h	24 h	48 h	24 h	48 h	24 h	48 h	24 h	48 h	24 h	48 h
CE	2.0	1.0	4.5	2.0	16.5	6.9	31.2	9.0	14.3	10.2	>100	>100
ChIVa	69.0	15.8	14.9	5.6	30.0	20.9	>100	50.3	>100	78.2	>100	>100
MIc	7.0	4.3	4.2	2.9	12.5	9.5	16.0	8.0	9.4	8.3	>100	>100
OA	13.0	8.3	>100	>100	51.5	10.7	48.7	24.5	25.6	18.5	>100	>100
DOX	3.4		1.5		1.0		1.1		4.0		1.7	

Abbreviations: calenduloside E (CE); chikusetsusaponin IVa (ChIVa); momordin Ic (MIc); oleanolic acid (OA); reference drug (DOX).

#### 2.2.5. Skin Panel

The cytotoxicity of oleanolic acid towards various melanoma cells is well documented [36,39,40,41]. However, the other compounds analyzed were poorly studied in this respect. Although the cytotoxic activity of CE and ChIVa against murine B16 cell line and their effects on the growth of B16F10 melanoma in tumor-bearing mice have been investigated [8], to our knowledge, the action of saponins (MIc, ChIVa, and CE) on human melanoma cells has not been analyzed so far. In our study, all the tested compounds showed a potent cytotoxic effect towards A375 (malignant melanoma) cells (IC_50_ = 1.6–2.2 μg/mL) after just 24 h of incubation (Table 5). The effect was time-dependent, resulting in IC_50_ values after 48 h of incubation in the range of 0.66–0.88 μg/mL. Most of the compounds tested also showed high activity against the WM793 line (primary stage 1 melanoma), with the exception of ChIVa, which had a moderate effect. It is noteworthy that each compound demonstrated higher cytotoxicity in this cancer cell line than the reference drug. In contrast, the activity of the compounds tested against the metastatic HTB140 line varied greatly. The highest cytotoxicity, superior to the reference drug, was observed for CE, whereas the other compounds displayed weaker effects and can be ranked in terms of activity as follows: MIc > ChIVa > OA. 

As the analyzed compounds affected the viability of the keratinocytes (HaCaT cell line), we decided to calculate the selectivity index (SI). A higher selectivity index indicates a greater effect against cancer cells and, at the same time, a weaker effect against normal cells, with a selectivity index > 2 indicating the selectivity of the compound [42,43]. Unfortunately, the most potent compounds tested in our study, CE and MIc, had low selectivity index values, similar to the reference drug. Therefore, any further studies on the activity of these compounds on skin cells would require the determination of the selective dose.

It is also worth noting that, recently, Luo et al. [44] demonstrated that the inhibition of HaCaT proliferation by MIc is mediated by the modulation of the Wnt/β-catenin pathway, and this effect could be exploited in the treatment of psoriasis.

**Table 5:** IC_50_ values and selectivity index (SI) values of CE, ChIVa, MIc, and OA on melanoma cell lines and keratinocytes.

IC_50_ [μg/mL] and (Selectivity Index (SI))								
	HTB-140		A375		WM793		HaCaT	
Comp.	24 h	48 h	24 h	48 h	24 h	48 h	24 h	48 h
CE	2.8 (1.8)	1.7 (1.1)	1.8 (2.9)	0.8 (2.6)	2.4 (2.1)	1.2 (1.6)	5.1	2.0
ChIVa	15.0 (0.5)	8.7 (0.4)	1.6 (4.6)	0.6 (2.3)	18.5 (0.4)	4.4 (0.7)	7.3	3.1
MIc	7.8 (0.7)	3.0 (0.9)	1.9 (2.7)	0.9 (2.0)	2.0 (2.6)	1.0 (2.7)	5.3	2.7
OA	51.4 (0.2)	29.5 (0.2)	2.2 (5.2)	0.9 (1.5)	2.8 (4.0)	1.2 (6.0)	11.2	7.5
DOX	5.7 (0.8)		0.6 (7.9)		>40.0		4.7	

Abbreviations: calenduloside E (CE); chikusetsusaponin IVa (ChIVa); momordin Ic (MIc); oleanolic acid (OA); reference drug (DOX).

### 2.3. Chemometric Analysis

A chemometric approach was used to investigate the overall impact of the compounds tested on the cell lines used in this study. Simple PCA models meeting the cross-validation criteria were constructed for the four compounds tested. The basic characteristics of these models are provided in Table S2. For each model, the loading scatterplot was created, and the corresponding correlation coefficients for the parameters were calculated. For example, Figure S10 shows a loading scatterplot for model 1. 

The hierarchical principal component analysis model that met the cross-validation criteria had two significant components with eigenvalues of 3.38 and 2.84, respectively. This model explained 77.7% of the variance in the eight new parameters obtained as principal components in the simple PCA models constructed for each tested compound, as described in the statistical approach section. The loadings of the first principal components in hPCA are shown in Figure S11. 

The first principal component in this model was positively loaded by the mutually strongly correlated four new parameters: t1c3, t1c2, t1c4, and t1c1. The second principal component was mainly positively loaded by another set of correlated parameters, i.e., t2c4 and t2c2, while negatively by t2c1 and t2c3. There were strong negative correlations between the members of the above two groups of parameters (Figure S11; Table 6). 

A comparison of the dependencies revealed by the hPCA model (Figure S11; Table 6) and the parameters playing the most important role in simple PCA models for individual compounds (Table S2) indicates that the following compounds are similar in their effects on the cell lines in relation to their concentrations: all the compounds at concentrations of 6 µg/mL, 10 µg/mL, and 20 µg/mL, saponins CE and ChIVa (4 µg/mL), and MIc (30 µg/mL) belong to this group. Compounds ChIVa and OA at the concentrations of −0.5 µg/mL, 50 µg/mL, and 100 µg/mL reacted quite differently but clearly similarly to each other. At the same concentrations (and additionally at a concentration of 1 µg/mL), CE and MIc had completely different effects on the cell lines. 

**Figure 4:**
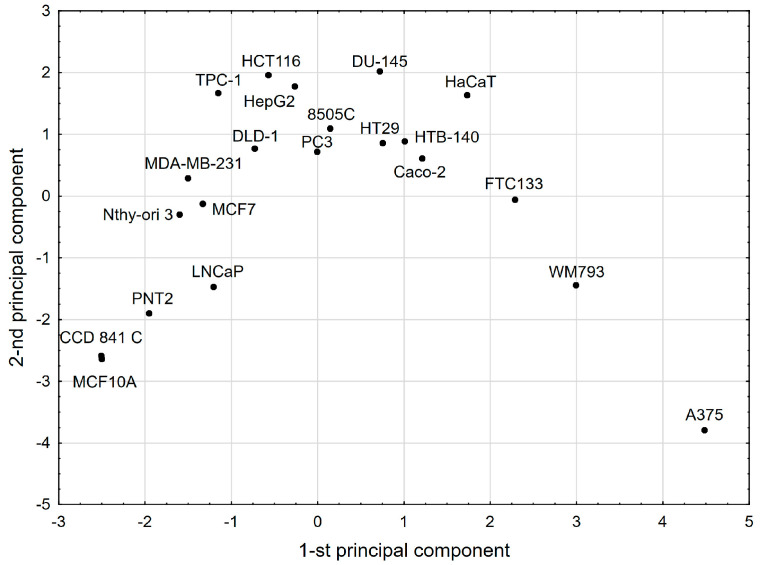
The score scatterplot of hPCA model (cell lines were depicted).

Figure 4 shows the cell lines tested in the space defined by the first two principal components of the hPCA model. The visual inspection of this plot revealed some similarities and differences between the cell lines. 

**Figure 5:**
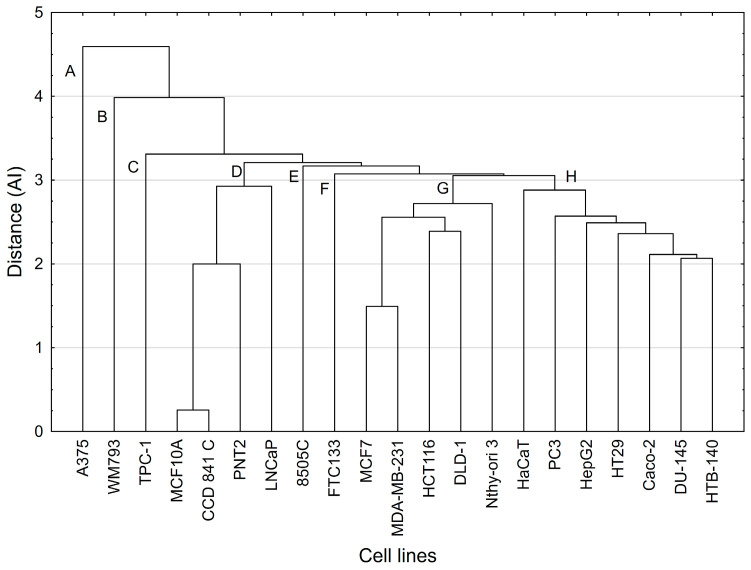
Dendrogram of similarity between different cell lines. Method of grouping: single linkage; function of the distance: Euclidean distance (subsequently identified clusters were marked with subsequent letters; further explanation in the text).

Essentially, the same clusters of the cell lines were confirmed by the CA method (Figure 5). The cell lines that were most distinct from the other lines were A375 and WM793 (Figure 4 and Figure 5). They also formed separate single-element clusters (A and B on Figure 5). Clear similarities between the cell lines were revealed for the members of cluster H (Figure 5): HTB-140, DU-145, Caco-2, HT-29, HepG2, PC3, and HaCaT. Another multi-element cluster, visible in both statistical methods, was cluster G (Figure 4 and Figure 5), containing MCF7, MDA-MB-231, HCT-116, DLD-1, and Nthy-ori 3. The greatest similarity in behavior occurred for the MCF10A and CCD 841 C lines located within cluster D. The remaining lines, TPC1, FTC133, and 8505C, were marked with some differences from the other lines. Interestingly, these are three thyroid cancer cell lines. 

The chemometric models summarize our observations that the cell lines in cluster D have characteristics that make them a group of cells that are not susceptible to the OA-type compounds analyzed. Given that this cluster comprises mainly normal cell lines, it reflects the selectivity of all the compounds tested. On the other hand, it is noteworthy that there are distinct single-element clusters, including the A375 cell line cluster (cluster A), which was found to be generally sensitive to all the analyzed oleanolic acid derivatives. Within the remaining clusters, the cancer cell lines with varying sensitivity to the compounds used predominate. This is related not only to the concentration of the tested substance but also to the sensitivity of a particular cell line to a specific compound structure. The observed similarities in the sensitivity of the cell lines to the tested compounds require further in-depth studies related to the cellular elements and metabolic pathways.

### 2.4. Structure–Activity Relationship

It is well known that the structure significantly influences the activity and potency of a compound. Although numerous studies have been published on the structure of saponins and their structure–activity relationships (SARs) [6,45], the data on this subject remain insufficient. However, the research involving comparative studies of the activity of OA-type saponins that contain a glucuronic acid (GlcA) residue in their sugar moiety is still incomplete, and the studies mainly focus on the ester derivatives of glucuronic acid [8,13,14,15,17,46,47,48,49,50]. Therefore, to fill the knowledge gap, we compared a group of OA-type saponins containing GlcA in the sugar part of the compound and their sapogenin, OA. Among the compounds analyzed were structures differing in the presence of the sugar moiety and its location, as well as in the number of monosaccharides in the glycone. In addition to the saponins isolated from *Ch. strictum* (CE and ChIVa), another derivative of oleanolic acid—momordin Ic (MIc)—and oleanolic acid (OA) itself were also included in the study (Figure 1). The cytotoxic activity of the selected OA analogues has not previously been directly compared

In this study, we analyzed a broad spectrum of cancer cell lines. The results showed that the observed effects are largely dependent on the susceptibility of the specific cell lines. However, the study also identified some regularities in the structure and activity relationships. It appears that the key structural element affecting the cytotoxic activity of OA derivatives is the presence of a free carboxyl group located at position C-17 (28-COOH) in the structure of oleanolic acid (OA). Blocking this group with a sugar chain significantly reduces or completely eliminates the activity (IC_50_ > 100 μg/mL; see Table 1, Table 2, Table 3, Table 4 and Table 5). This trend is evident in most cell lines when comparing the cytotoxicity of the bidesmoside (ChIVa) with the corresponding monodesmoside (CE), as well as saponin MIc. Our observations confirm the data from previous studies comparing the activity of CE with ChIVa against human colon WiDr, Lovo cell lines, and mouse B16F10 melanoma cells [8,14]. The results obtained are also consistent with other studies on oleanolic acid derivatives with different sugar moieties [14,17,50,51,52,53,54]. For some cell lines, such as WM-793, 8505C, TPC-1, Caco-2, DLD-1, and HTC-116, compound ChIVa showed even weaker activity than its sapogenin, OA. Interestingly, an exception to this trend was observed in a melanoma cell line (A375), where the saponin displayed comparable or slightly higher activity than the other compounds analyzed, including CE. It is worth noting that, although the free carboxyl group at the core of the oleanolic acid structure is crucial for activity, it is not the only element affecting the observed cytotoxic effect. In the present study, we also confirmed that the two analyzed oleanolic acid monodesmosides generally showed significantly higher activity against the cell lines tested than their sapogenin (OA). The analysis showed that bidesmoside (ChIVa) also exhibited higher activity than OA against the HTB-140, A375, HaCaT, FTC133, HT-29, and PC3 cell lines. This observation suggests that the presence of the sugar moiety attached to the oleanolic acid core is particularly important for the cytotoxic activity of saponins against these human cell lines since even blocking the carboxyl group by attaching glucose did not eliminate the saponin activity.

As shown in Table 1, Table 2, Table 3, Table 4 and Table 5, among the compounds analyzed in this study, monodesmosides with a sugar chain at the C-3 position of the oleanolic acid exhibited the highest activity. These structures differed only in the presence or absence of an additional xylose residue in the sugar chain. Our study showed that the activity of the analyzed OA monodesmosides is closely related to the specific cell line tested. The activity of both saponins, CE and MIc, was comparable against the WM793, HT29, PC-3, A375, and HaCaT cell lines. The differences in the activities of these saponins were evident against the other lines analyzed. CE showed significantly higher cytotoxic activity than its analogue, particularly against the thyroid cancer cell lines (FTC133, 8505C, and TPC-1), colon cancer (Caco-2) cells, and melanoma HTB140 cells. In turn, MIc, with a two-sugar chain with xylose, demonstrated higher cytotoxic activity than CE against the cancer DLD-1, HCT116, and MDA-MB-231 cell lines and slightly higher against HepG2 and Du-145 cells.

Several published reports indicate that the xylose residue may be an important component of the sugar chain, affecting the anticancer activity [46,47].

**Figure 6:**
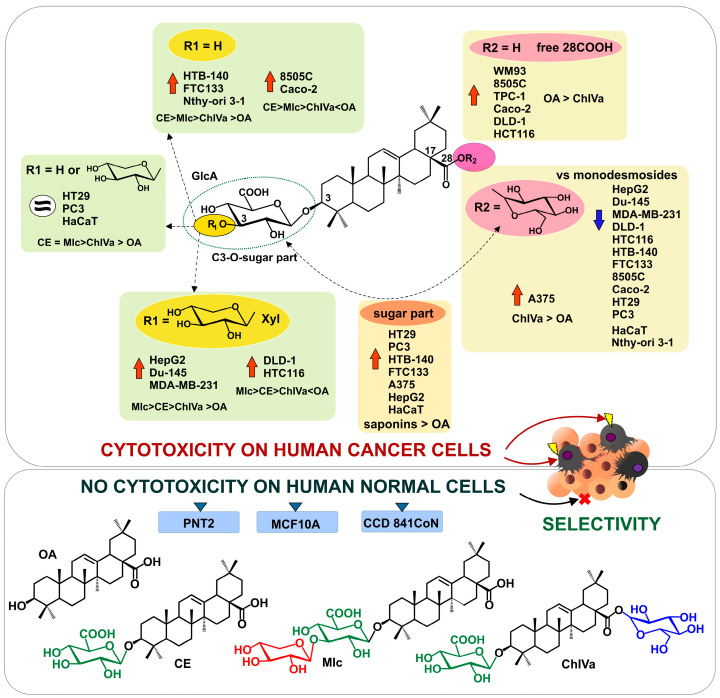
The most important structure–activity correlations for cytotoxic activity of analyzed saponins. Abbreviations: ↑—increased activity; ≈—similar activity; ↓—decreased activity; calenduloside E (CE); chikusetsusaponin IVa (ChIVa); momordin Ic (MIc); oleanolic acid (OA).

Some studies also suggest that an important structural feature is the presence of a disaccharide chain with a 1→2 linkage between the sugars and a free hydroxyl group located at the C-3′ of the monosaccharide attached to C-3 of the aglycone. In contrast, a 1→3 disaccharide moiety reduces the activity of the structure [52]. However, these observations refer to oleanane-type saponins, which are not structurally similar to those analyzed in this study. Nevertheless, it is worth noting that, in the structure of momordin Ic, xylose is attached to GlcA via a 1→3 bond, which may explain its lower activity compared to calenduloside E. As we did not investigate other possible combinations of the Xyl and GlcA links, we can only conclude that the elongation of the sugar chain by an additional xylose residue (at C3′ of GlcA) modifies the effect of the compounds on specific types of human cells. The summary of the structure–activity observations derived from this study is presented in Figure 6. 

## 3. Materials and Methods

### 3.1. Chemicals and Reagents

Methanol, chloroform, ethyl acetate, butanol, 2-propanol, and acetone were obtained from CHEMPUR (Gliwice, Poland). Hydrochloric acid was obtained from Avantor Performance Materials Poland S.A. (Gliwice, Poland). All reagents were of analytical grade. Momordin Ic (phyproof^®^ Reference Substance, purity ≥98.0% (HPLC), oleanolic acid (≥97%), D-glucose, D-galactose, L-arabinose, D-xylose, glucuronic acid, HPLC grade formic acid, lactic acid, and monosodium phosphate were purchased from Sigma-Aldrich (St. Louis, MO, USA). HPLC-grade methanol was purchased from Merck (Darmstadt, Germany). Water was prepared using a Milli-Q system (Millipore Corp., Bedford, MA, USA).

### 3.2. General Experimental Procedures

Medium-pressure liquid chromatography (MPLC) was performed on a Sepacore apparatus equipped with a C-615 Pump Manager (BÜCHI Labortechnik AG, Flawil, Switzerland). MPLC was carried out on NP: silica gel 230–400 mesh (Sigma-Aldrich, Darmstadt, Germany) and RP: reverse phase silica gel (LiChroprep, RP-18 (40–63 μm), Merck, Darmstadt, Germany). 

Preparative high-pressure liquid chromatography (HPLC) was performed on a KNAUER Azura system (Knauer GmbH, Berlin, Germany): P 4.1S pump and a UV/VIS K-2600 detector (detection at 210 nm). Chromatographic separations were carried out using the Gemini^®^ 5 µm NX-C18 110 Å LC column 250 × 10 mm (Phenomenex, Torrance, CA, USA) equipped with SecurityGuard SemiPrep cartridges Gemini-NX C18 10 × 10 mm precolumn. 

Open-column chromatography (CC) was carried out on NP: silica gel (230–400 mesh; Sigma-Aldrich, Germany) using a glass column (350 × 25 mm). 

Thin-layer chromatography (TLC) was performed on NP: silica gel 60 plates (Merck, Germany) and RP-18 F254S silica gel 60 plates (Supelco, Darmstadt, Germany). Detection: 25% solution of H_2_SO_4_ in methanol was used as spraying reagent, and visualization occurred after heating the plate at 120 °C for 4 min on a TLC plate heater (CAMAG, Muttenz, Switzerland). 

LC–MS analysis was performed on UPLC/MS Waters ACQUITY TQD (Waters Corporation, Milford, MA, USA) apparatus operated in the negative and positive electrospray ionization modes. The Acquity UPLC bridged ethyl hybrid -BEH C18 column (100 × 2.1 mm, and particle size of 1.7 μm), equipped with Acquity UPLC BEH C18 VanGuard precolumn (2.1 × 5 mm, and particle size of 1.7 μm) was used for chromatographic separations under conditions described previously [55]. MS detection settings of Waters TQD mass spectrometer were also described previously [55].

NMR spectra (1D (^1^H NMR—500 MHz and DEPTQ—125 MHz) and 2D (HSQC, F2-coupled HSQC, H2BC, HMBC, COSY, TOCSY, and T-ROESY) were recorded in the mixture of pyridine-d_5_/D_2_O (250/10) with 0.2% TFA on a BrukerAvance III HD Ascend-500 spectrometer (Bruker BioSpin, Rheinstetten, Germany), equipped with 5 mm 1H{109Ag-31P} broad-band inverse (BBI) probe; coupling constants are reported in Hz. Chemical shifts (δ) are provided in ppm. Spectra were analyzed using ACD/Labs 1D NMR Processor 12.0 (Academic Edition)(Advanced Chemistry Development, Inc, Toronto, Ontario, Canada) and CARA 1.9.1.4. - Computer Aided Resonance Assignment (CARA Definition Team (CDT), free software licensed under the GNU General Public Licence (GPL)) 

The cytotoxicity study was conducted using a Microplate Reader (BioTek Instruments Inc., Winooski, VT, USA) with Gen 5 software.

### 3.3. Plant Material

*Chenopodium strictum* Roth was collected from natural habitat in July 2019, near Cracow, Poland (50°00′42.7″ N 19°59′42.6″ E). Species identity was confirmed by a botanist from the Department of Pharmacognosy UJCM, Cracow, Poland. The voucher specimen (No. Ch_str/2019_B) has been deposited at the Department of Pharmacognosy, Pharmaceutical Faculty, Medical College, Jagiellonian University, Cracow, Poland. 

Plant material was divided into parts (roots, leaves, stems, and fruits) and dried under steady, controlled conditions (at 23 °C in air-conditioned room, in the dark) to a constant weight. The plant material was cut and ground to a fine powder using a mechanical laboratory mill (BOSCHMKM6003, BSHGmbH, Munich, Germany), and then kept in airtight containers.

### 3.4. Extraction and Isolation

The samples of powdered plant material (roots—2 g, leaves—2 g, fruits—2 g, and stems—2 g) were placed in a round bottom flasks and extracted sequentially with chloroform (plant material/solvent ratio—DSR 1:10, 2 times for 1 h) and methanol (DSR 1:10, 2 times for 2 h) by heat reflux extraction (under cooler with the use of a heating mantle). The combined MeOH extracts were concentrated under reduced pressure on a rotary evaporator to yield residues from roots (215 mg), leaves (427 mg), stems (317 mg), and fruits (147 mg). The dried crude extracts were subjected to thin-layer chromatography (TLC) using the conditions developed and described previously: silica gel, CHCl_3_-CH_3_OH-H_2_O (20:12:2 *v*/*v*), 25% methanolic H_2_SO_4_ + heating (120 °C/4 min) [32]. Preliminary TLC investigation showed a presence of saponins in the roots of *Ch. strictum* (Figure S1). To isolate the saponins, extract from a larger sample of *Ch. strictum* roots (250 g) was prepared. Extraction was performed using the conditions described above for a small sample of the plant material. The methanol extract was concentrated under reduced pressure using a rotary evaporator, and 22.7 g of a brown viscous residue was obtained, which was suspended in water and extracted twice with n-BuOH (100 mL). The n-butanol extract was evaporated to dryness under reduced pressure on a rotary evaporator (temp. 50–60 °C) to yield 15.5 g. Portions of the n-BuOH extract (2.0 g) were fractionated by column chromatography (CC, column 25 × 350 mm; silica gel 230–400 mesh) using the isocratic solvent system (CHCl_3_-CH_3_OH-H_2_O (20:12:2 *v*/*v*)). Fractions were collected and combined based on TLC examination (silica gel 60 plates, CHCl_3_-CH_3_OH-H_2_O (20:12:2 *v*/*v*). 

CC chromatography yielded 14 pooled fractions (Fr/1-Fr/14), of which fractions F/4 and F/8 were further subjected to medium-pressure liquid chromatography (MPLC) on silica gel (230–400 mesh). The following conditions were used to fractionate Fr/4: MPLC, column 12 × 150 mm, flow rate 3 mL/min); isocratic solvent system: CHCl_3_-CH_3_OH-H_2_O (23:12:2 *v*/*v*). Fractions were combined based on TLC analysis (silica gel, CHCl_3_-CH_3_OH-H_2_O (23:12:2 *v*/*v*), 25% methanolic H_2_SO_4_ + heating, to yield 6 major fractions (Fr4/A-Fr4/F). The fraction Fr4/C rich in compound **1** (R_f_ = 0.51) was further separated by RP-MPLC chromatography (using solvent system: CH_3_OH-H_2_O (7:1.5 *v*/*v*) in conditions previously described [32] to yield seven fractions (Fr4C/1-Fr4C/7), of which fraction Fr4C/4 constituted compound **1** (21 mg), while fractions Fr4C/5 and Fr4C/6 were combined (31 mg) and further purified using a preparative RP-HPLC technique. The following chromatographic conditions were used: Phenomenex Gemini^®^ 5 µm NX-C18 110 Å LC column 250 × 10 mm (Phenomenex, Torrance, CA, USA); solvent system: CH_3_OH-H_2_O-HCOOH (8:2:0.01 *v*/*v*/*v*); flow rate: 2 mL/min; UV detection at 210 nm), which led to isolation of 10 mg of compound **1**. Fractions containing compound **1**, obtained by both MPLC and preparative HPLC, were controlled using UPLC-ESI-MS method under conditions described previously [55] and pooled based on similarity and purity. As a result, the separation process yielded 36 mg of compound **1**. 

Fraction Fr/8 was chromatographed by MPLC (silica gel 60, column 12 × 150 mm, flow rate 2.5 mL/min) using the following solvent system: CHCl_3_-CH_3_OH-H_2_O (30:25:5 *v*/*v*) to yield 7 fractions (Fr8/A-Fr8/G). 

The Fr8/C and Fr8/D were combined based on TLC similarity (silica gel, CHCl_3_-CH_3_OH-H_2_O (30:25:5 *v*/*v*); 25% methanolic H_2_SO_4_ + heating (120 °C/4 min) and further purified by RP-MPLC (using solvent system: CH_3_OH-H_2_O (7:3) in conditions previously described [32]. The separation process using following conditions yielded 24 mg of compound **2**.

### 3.5. Structure Elucidation

NMR spectra were recorded on a BrukerAvance III HD Ascend-500 spectrometer (Bruker BioSpin, Rheinstetten, Germany) (see Section 3.2, General Experimental Procedures). Spectra were analyzed using ACD/Labs 1D NMR Processor 12.0 (Academic Edition) and CARA 1.9.1.4. (Computer Aided Resonance Assignment).

LC–MS analysis was performed on UPLC/MS Waters ACQUITY TQD (Waters Corporation, Milford, MA, USA) apparatus (see Section 3.2, General Experimental Procedures).

Acid hydrolysis of isolated saponins (compound **1** and **2**) was performed under the conditions described previously [56]. The following sugar standards were used for analysis: D-galactose R_f_ = 0.26, D-glucose R_f_ = 0.38; L-arabinose R_f_ = 0.45; D-xylose R_f_ = 0.6, glucuronic acid lacton R_f_ = 0.80. 

#### 3.5.1. Compound **1** (CE): Oleanolic Acid-3-*O*-*β*-d-Glucuronopyranoside (Caleduloside E)

White powder: ESI MS (negative and positive ion modes)—data previously described [32]. Acidic hydrolysis: sugar spot R_f_ = 0.81. For ^1^H and ^13^C NMR spectroscopic data, see Table S1 (Supplementary Materials).

#### 3.5.2. Compound **2** (ChIVa): 3-O-β-d-Glucuronopyranosyl Oleanolic Acid 28-O-β-d-Glucopyranosyl Ester (Chikusetsusaponin IVa)

White powder: ESI MS (negative and positive ion modes)—data previously described [32]. Acidic hydrolysis: sugar spots: R_f_ = 0.38, R_f_ = 0.80. For ^1^H and ^13^C NMR spectroscopic data, see Table S1 (Supplementary Materials). 

### 3.6. Cell Culture

Cell lines used in the study were grouped as follows: skin panel (melanomas: HTB140, derived from metastatic site: lymph node, ATCC Hs 294 T; malignant A375, ATCC CRL-1619; stage I primary WM793, RRID:CVCL 8787; skin keratinocytes HaCaT); prostate panel (prostate carcinomas: Du145, derived from metastatic site: brain, ATCC HTB-81; grade IV PC3, derived from metastatic site: bone, ATCC CRL-1435, LNCaP, derived from the metastatic site: lymph node, ATCC CRL-1740; prostate epithelial, PNT2, ECACC 95012613); gastrointestinal panel (colorectal adenocarcinomas: Caco2, ATCC HTB-37, HT29, ATCC HTB-38, DLD-1, ATCC CCL-221, HCT116, ATCC CCL-247, and colon epithelial cells CCD 841 CoN, ATCC CRL-1790; hepatocellular carcinoma HepG2, ATCC HB-8065); thyroid panel (follicular thyroid carcinoma FTC133, ECACC 9406090, undifferentiated thyroid carcinoma 8505C, ECACC 94090184, papillary thyroid carcinoma TPC-1, SCC147, normal thyroid follicular epithelial Nthy-ori 3–1, ECACC 90011609); and breast panel (ER-positive breast adenocarcinoma MCF7, ATCC HTB-22; ER-negative breast adenocarcinoma MDA-MB-231, ATCC HTB-26; breast epithelial MCF10A, ATCC CRL-10317). Cells were grown under standard conditions (37 °C, 5% CO_2_, relative humidity), and culture media (DMEM/F12 for PNT2, WM 793, HT29, PC3, FTC133, 8505 C, MDA-MB-231, TPC-1; DMEM Low Glucose for DU145; DMEM High Glucose for HTB140, A375, HaCaT; MEM with NEAA for Caco2 and MCF7; RPMI1640 with sodium pyruvate for LNCaP; DMEM/F12 with 20 ng/mL epidermal growth factor (EGF), 10 μg/mL insulin, 0.5 μg/mL hydrocortisone, 100 ng/mL cholera toxin) for MCF10A) supplemented with 10% fetal bovine serum (FBS) or 5% donor horse serum for MCF10A, and 1% antibiotics solution (10,000 U penicillin and 10 mg streptomycin/mL) supplemented with 10% fetal bovine serum (FBS) and 1% antibiotics solution (10,000 U penicillin and 10 mg streptomycin/mL), as previously described [57,58]. All cell lines, culture media, and supplements were from Sigma-Aldrich (Germany).

### 3.7. Cell Viability Assay

The cells were seeded in 96-well plates (1.5 × 10^4^ cells/well). After 24 h, fresh medium was added, containing the tested compounds (0.5–100 μg/mL) and incubated for 24 or 48 h. Cell viability was determined by MTT assay, according to the manufacturer’s instructions, as described previously [58]. Each experiment was performed in triplicate. The absorbance was measured at 570 nm using a Biotek Synergy microplate reader (BioTek Instruments Inc., Winooski, VT, USA). Cell viability was expressed as a percent of control (untreated cells). Doxorubicin was used as a reference drug.

### 3.8. Selectivity Index

The selectivity index (SI) was calculated from the following formula:

SI = IC_50_ of compound in a normal cell line/IC_50_ of compound in cancer cell line.

IC_50_, half maximal inhibitory concentration, is a concentration required to kill 50% of the cells.

### 3.9. Chemometric Analysis

For each tested compound, a simple principal component analysis (PCA) model was constructed, in which cell lines were the objects and different concentrations of the test compound were the parameters. All parameters in all PCA models were mean-centered and scaled to unit variance. The parameters with large absolute values of their coordinates (>0.3) on the first two principal components in the PCA model were assumed to determine the axes of the new coordinate system in PCA to the greatest extent. 

To express the strength of bivariate associations between such parameters, the cosines of the corresponding angles (i.e., correlation coefficients) were calculated. The “corresponding angle” means the angle determined by the two lines connecting the origin with coordinates of both parameters on the PCA loadings plot. The parameters were considered negatively correlated if their loadings within the PCA model showed the opposite signs; otherwise, they were considered positively correlated. Such association indicates only a similar effect of different concentrations of the tested compound on the cell lines used. 

In the superior hierarchical PCA (hPCA) model, the first two principal components of each simple PCA model (previously constructed for the four tested compounds) were used as independent parameters. 

A cluster analysis (CA) approach was used to test whether clusters of cell lines that appeared in the hPCA scores plot would be confirmed by another multivariate statistical method and, if so, what the possible reason was for this. Cluster analysis of results of cell lines (i.e., t-scores) obtained on the first three principal components of each of the above-mentioned four simple PCA models used single linkage procedure as a method of grouping and Euclidean distance as a function of the distance. 

### 3.10. Data Analysis

The results were expressed as mean (±SD). Data were analyzed using Statistica v.13.3 (StatSoft, Tulsa, OK, USA). One-way analysis of variance (ANOVA) and the post hoc Tukey multiple comparison test were used. The probability level of *p* < 0.05 was considered statistically significant. Statistical analysis (PCA, CA) was conducted using packages STATISTICA v.13 (TIBCO Software Inc., Palo Alto, CA, USA) and SIMCA-P v.9 (Umetrics, Umeå, Sweden). The IC50 values in the cytotoxic assay were determined using Origin Pro 2020b (OriginLab Corporation, Northampton, MA, USA). Chemical structures of the compounds were illustrated with ChemDraw 19.0 software. Graphs were created with Excel 365 (Microsoft Office), and illustrations were designed using CorelDraw 2021.5.

## 4. Conclusions

Two known saponins, calenduloside E (CE) and chikusetsusaponin IVa (ChIVa), have been isolated from the roots of *Chenopodium strictum* Roth. Thus, our study revealed that *Chenopodium strictum* should be considered as a novel source of these oleanolic acid (OA)-type saponins. To the best of our knowledge, this is the first report on saponins in this plant species and constitutes a contribution to the general knowledge of the chemical composition of the Amaranthaceae family. 

Both isolated saponins and the structurally similar momordin Ic (MIc), as well as oleanolic acid (OA), were found active in a cytotoxic assay, but the observed effect depended on the structure of the compound and the susceptibility of the human cancer cell line. 

It appears that the key structural elements responsible for the strong cytotoxic activity against most cancer cell lines are the free carboxyl group (28COOH) in the structure of sapogenin (OA) and the presence of a sugar moiety in the molecule. Although the activity depends on the susceptibility of the specific cell line, monodesmosides containing a sugar moiety with glucuronic acid at the C3 position of OA have a stronger effect than bidesmoside or OA alone.

The results of our study enable suggesting that the presence of an additional xylose in the sugar chain affects the activity towards cancer cells, but this effect strictly depends on the specific cell line. The presence of an additional xylose residue at the C’3 position of the glucuronic acid in the sugar moiety suppressed the cytotoxicity against HTB-140, FTC133, and 8505C,Caco-2 cells while increasing the activity against DLD-1, HTC116, MDA-MB-231, and HepG2 cells compared to the effect of the compound without an extended sugar moiety. The obtained data indicate the importance of SAR screening against a large number of different cancer cells as incomplete conclusions regarding the overall activity may be drawn based on analyses of only one or a few cell lines.

The findings of this study revealed that OA-saponins bearing GlcA, especially CE and MIc, due to their cytotoxic potential and selectivity, are good candidates for further research on thyroid, breast, prostate, and gastrointestinal cancers. Nevertheless, further experiments in in vivo models are needed to confirm these in vitro observations. On the other hand, although the tested oleanolic acid derivatives, particularly CE, revealed high cytotoxicity towards melanoma cells, they did not act selectively. Therefore, while their potential against skin cancers is promising, our research indicates the need to explore ways to reduce their toxicity towards normal skin cells.

## Figures and Tables

**Table 6:** Correlation coefficients for the pairs of parameters based on hPCA model.

	Correlated Parameters								
		t1c1	t1c2	t1c3	t1c4	t2c1	t2c2	t2c3	t2c4
**Correlated parameters**	**t1c1**		0.888	0.991	0.867				
	**t1c2**	0.888		0.941	0.999				
	**t1c3**	0.991	0.941		0.925				
	**t1c4**	0.867	0.999	0.925					
	**t2c1**						−0.972	1.000	−0.986
	**t2c2**					−0.972		−0.969	0.998
	**t2c3**					1.000	−0.969		−0.984
	**t2c4**					−0.986	0.998	−0.984	

## Data Availability

Data are contained within the article or Supplementary Materials.

## Supplementary Materials

The following supporting information can be downloaded at https://www.mdpi.com/article/10.3390/molecules29163794/s1, Figure S1. TLC chromatogram of methanol extracts from leaves (1), stems (2), roots (3), and seeds (4) of *Ch. strictum*. Table S1. ^1^H (500 MHz) and ^13^C DEPT Q (125 MHz) NMR spectral data (δ ppm) for compound **1** and **2** (pyridine-d5/D2O 250:10 with 0.2% trifluoroacetic acid); Figure S2. ^1^H (500 MHz) spectrum of compound **1**; Figure S3. ^13^C DEPT Q (125 MHz) spectrum of compound **1**; Figure S4. ^1^H (500 MHz) spectrum of compound **2**; Figure S5. ^13^C DEPT Q (125 MHz) spectrum of compound **2**; Figure S6. UPLC (PDA) chromatogram of compound **1**; Figure S7. UPLC (PDA) chromatogram of compound **2**; Figure S8. ESI-TQD-MS and MS/MS spectra (negative and positive ion mode) of compound **1**; Figure S9. ESI-TQD-MS and MS/MS spectra (negative and positive ion mode) of compound **2**. Supplementary Note 1: Structure elucidation. Table S2. The basic features of PCA models constructed for compounds **1**–**4**. Figure S10. The loading scatterplot for model **1**. Figure S11. The loading scatterplot for hierarchical principal component analyses model.
