# Supplementary material for: Selectivity Screening and Structure–Cytotoxic Activity Observations of Selected Oleanolic Acid (OA)-Type Saponins from the Amaranthaceae Family on a Wiade Panel of Human Cancer Cell Lines
Source: Molecules. 2024 Aug 10;29(16):3794. doi: 10.3390/molecules29163794 (PMC11357256; doi:10.3390/molecules29163794)
Supplement: Supplementary file 1 [file molecules-29-03794-s001.zip › molecules-3138110-supplementary.pdf]

Supplementary Materials

Selectivity screening and structure-cytotoxic activity observations of selected oleanolic acid (OA)-type saponins from the Amaranthaceae family on a wide panel of human cancer cell lines

Karolina Grabowska [1], Agnieszka Galanty [1,*] Łukasz Pecio [2,3], Anna Stojakowska [4], Janusz Malarz [4], Paweł Żmudzki [5,6], Paweł Zagrodzki [7] and Irma Podolak [1]

[1] Department of Pharmacognosy, Jagiellonian University Medical College, 9 Medyczna Str., 30-688 Cracow, Poland
[2] Department of Biochemistry and Crop Quality, Institute of Soil Science and Plant Cultivation–State Research Institute, ul. Czartoryskich 8, 24-100 Puławy, Poland
[3] Department of Chemistry of Natural Products, Medical University of Lublin, Lublin, 20-093,Poland
[4] Maj Institute of Pharmacology, Polish Academy of Sciences, Smętna Street 12, 31-343 Kraków, Poland
[5] Department of Medicinal Chemistry, Jagiellonian University Medical College, 9 Medyczna Str., 30-688 Cracow, Poland
[6] Center for the Development of Therapies for Civilization and Age-Related Diseases, Jagiellonian University Medical College, Skawińska 8, 31-066 Krakow, Poland
[7] Department of Food Chemistry and Nutrition, Medical College, Jagiellonian University, 9 Medyczna, 30-688 Kraków, Poland
Correspondence: agnieszka.galanty@uj.edu.pl;

Abstract: Plants from the Amaranthaceae family are a source of oleanolic acid (OA)-type saponins with cytotoxic activity. Two known OA-type saponins: calenduloside E and chikusetsusaponin IVa, were isolated from the roots of *Chenopodium strictum* Roth. Their structures were confirmed using MS and NMR techniques. This constitutes the inaugural report of saponins in *Ch. strictum*. Both isolated saponins and structurally similar compounds: momordin Ic and OA, were compared for their cytotoxicity against various cancer and normal cell lines (including skin, breast, thyroid, gastrointestinal, and prostate panels). Their effects were dose- and time-dependent, varying with the specific cell line and compound structure. A chemometric approach demonstrated the effects of compounds on cell lines. The study discusses structure-activity observations. Key structural elements for potent cytotoxic activity included the free carboxyl group-28COOH in the sapogenin structure (OA) and the presence of a sugar moiety. Monodesmosides with glucuronic acid (GlcA) at the C3 position of OA were generally more cytotoxic than bidesmosides or OA alone. The addition of xylose in the sugar chain modified the activity towards cancer cells, depending on the specific cell line. OA-type saponins with GlcA (particularly calenduloside E and momordin Ic) represent a promising avenue for further investigation as potential anti-cancer agents.

Keywords: *Chenopodium strictum*, calenduloside E, chikusetsusaponin IVa, momordin Ic, cytotoxic, structure-activity, saponins

Table of contents:

Figure S1. TLC chromatogram of methanol extracts from leaves (1), stems (2), roots (3), and seeds (4) of *Ch. strictum*.
Table S1. ^{1}H (500 MHz) and ^{13}C DEPT Q (125 MHz) NMR spectral data (δ ppm) for compound **1** and **2** (pyridine-d_5/D_2O 250:10 with 0.2% trifluoroacetic acid).
Figure S1. ^{1}H (500 MHz) spectrum of compound 1.
Figure S2. ^{13}C DEPT Q (125 MHz) spectrum of compound 1.
Figure S3. ^{1}H (500 MHz) spectrum of compound 2.
Figure S4. ^{13}C DEPT Q (125 MHz) spectrum of compound 2.
Figure S5. UPLC (PDA) chromatogram of compound 1
Figure S6. UPLC (PDA) chromatogram of compound 2.
Figure S7. ESI-TQD-MS and MS/MS spectra (negative and positive ion mode) of compound 1.
Figure S8. ESI-TQD-MS and MS/MS spectra (negative and positive ion mode) of compound 2.
Supplementary note 1. Structure elucidation.
Table S2. The basic features of PCA models constructed for compounds 1-4.
Figure S9. The loading scatter plot for model 1 (the numbers next to the points indicate the concentration of compound 1 in subsequent solutions, μg/mL).
Figure S10. The loading scatter plot for hierarchical principal component analyses model (the symbol t1c1 denotes first principal component in PCA model for CE; the symbol t1c2 denotes second principal component in PCA model for CE; etc.).

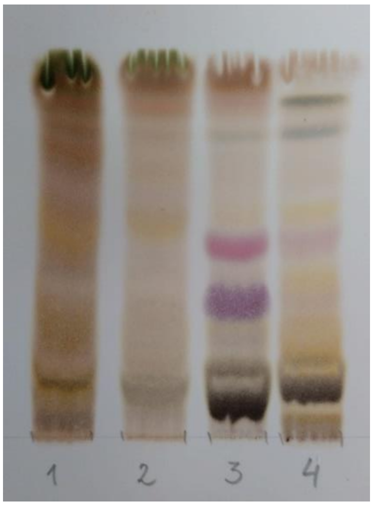

Figure S1. TLC chromatogram of methanol extracts from leaves (1), stems (2), roots (3), and seeds (4) of *Ch. strictum*.
[silica gel, solvent system: $CHCl_3$-CH_3OH-H_2O (20:12:2 v/v), visualisation: 25% methanolic H_2SO_4 + heating H_2SO_4 (120°C/4 min.)]

Table S1. ^{1}H (500 MHz) and ^{13}C DEPT Q (125 MHz) NMR spectral data (δ ppm) for compound **1** and **2** (pyridine-d_5/D_2O 250:10 with 0.2% trifluoroacetic acid).

	**Compound 1**		**Compound 2**	
**No.**	**δC**	**δH (*J* in Hz)***	**δC**	**δH (*J* in Hz)***
1	39.07	1.38;<br>0.81 t (12.0)	39.12	1.36 d (13.0);<br>0.82 t (12.0)
2	26.80	2.24, d (13.1);<br>1.82	26.74	2.28, d (12.5);<br>1.82, q (12.5)
3	89.70	3.32, dd (11.7, 4.3)	89.71	3.33, dd (11.9, 4.5)
4	39.87	-	39.86	-
5	56.26	0.74, d(11.9)	56.27	0.75, d(11.8)
6	18.89	1.47; 1.26	18.93	1.41; 1.24
7	33.61	1.45; 1.26	33.56	1.42; 1.30
8	40.15	-	40.31	-
9	48.41	1.59, t (8.9)	48.41	1.56, t (8.8)
10	37.36	-	37.34	-
11	24.17	1.85	24.20	1.83
12	123.00	5.44, t (3,8)	123.3	5.35, t (3,6)
13	145.22	-	144.54	-
14	42.58	-	42.55	-
15	28.73	2.12; 1.18	28.65	2.28; 1.18, d (13.6)
16	24.09	2.11; 1.94	23.82	2.07, t (12.8);<br>1.94, d (12.8)
17	47.10	-	47.46	-
18	42.40	3.25, dd (13.9, 4.6)	42.17	3.15, dd (14.0, 4.7)
19	46.92	1.79, t (13.6);<br>1.27	46.68	1.75, t (13.7);<br>1.24, d (13.3)
20	31.37	-	31.18	-

21	34.64	1.42; 1.18	34.41	1.32; 1.08
22	33.61	1.99; 1.18	32.94	1.81; 1.72
23	28.67	1.26, s	28.68	1.25, s
24	17.39	0.94, s	17.41	0.94, s
25	15.85	0.77, s	15.94	0.79, s
26	17.82	0.94, s	17.88	1.04, s
27	26.61	1.28, s	26.54	1.24, s
28	180.69	-	177.03	-
29	33.71	0.94, s	33.57	0.89, s
30	24.20	0.98, s	24.09	0.86, s
3-O-*β*-D-GlcA				
1	107.14	4.85, d (7.7)	107.00	4.82, d (7.7)
2	75.61	4.00, t (8.3)	75.60	4.00, t (8.4)
3	78.41	4.22, t (8.6)	78.52	4.19, t (8.5)
4	73.90	4.34	73.92	4.26
5	77.00	4.41	76.80	4.29
6	nd		nd	
28-O-*β*-D-Glc				
1			96.12	6.23, d (8.1)
2			74.33	4.15, t (8.4)
3			79.00	4.24, t (9.1)
4			71.43	4.28, t (9.1)
5			79.58	3.98
6			62.53	4.40, dd (12.0, 2.5);<br>4.32, dd (12.0, 4.6)

*Overlapping signals are reported without designated multiplicity.

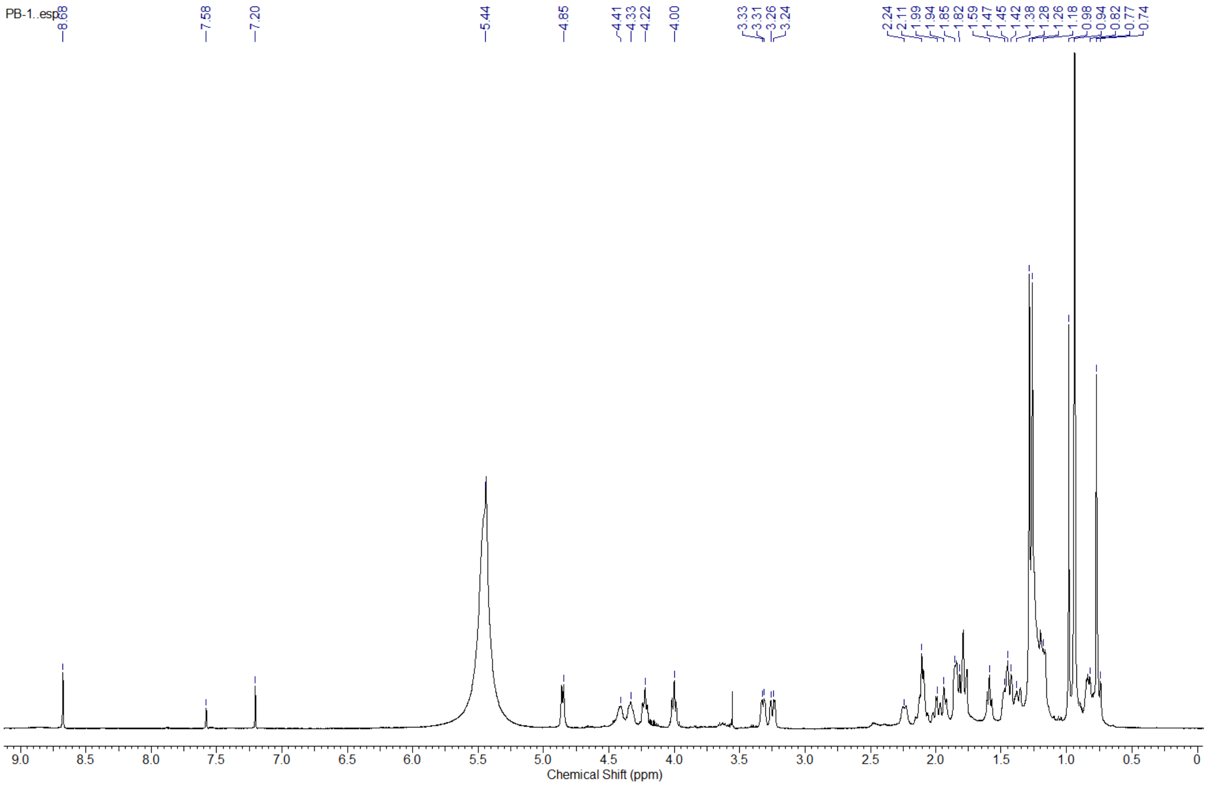

Figure S2. ^{1}H (500 MHz) spectrum of compound 1.

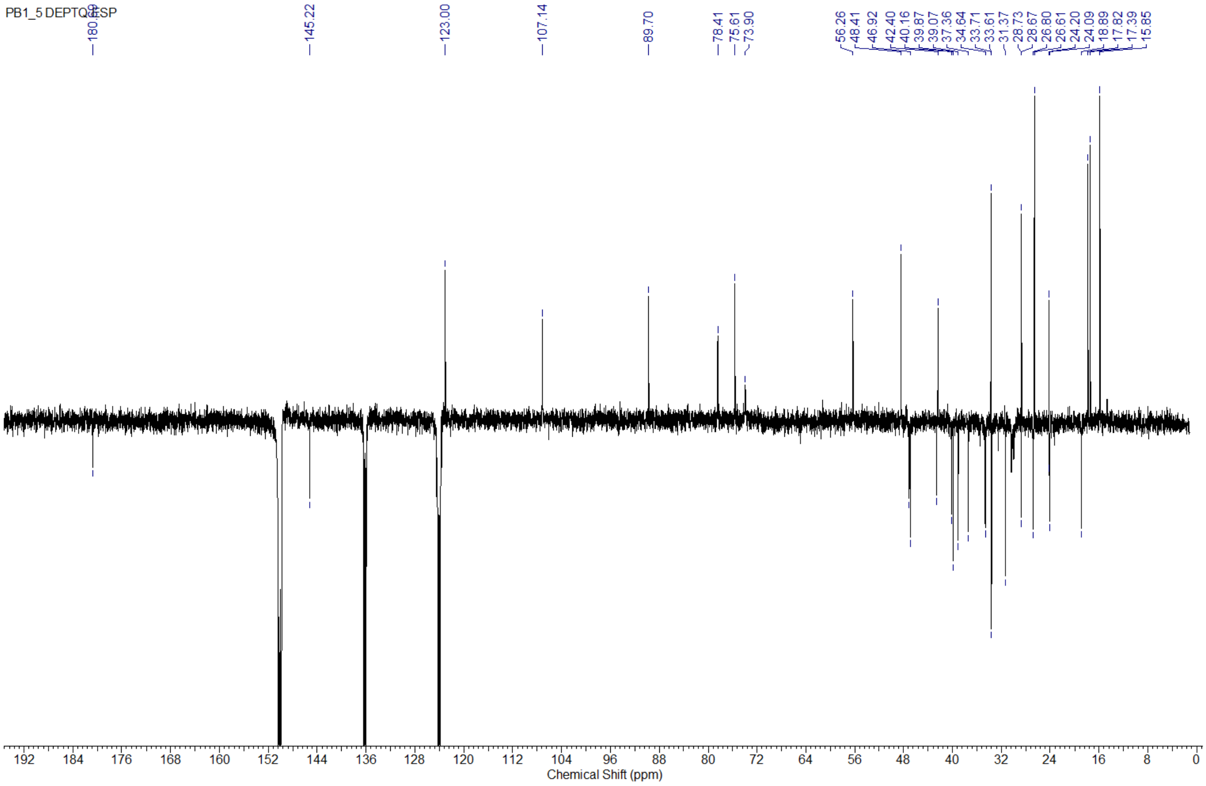

Figure S3. ^{13}C DEPT Q (125 MHz) spectrum of compound 1.

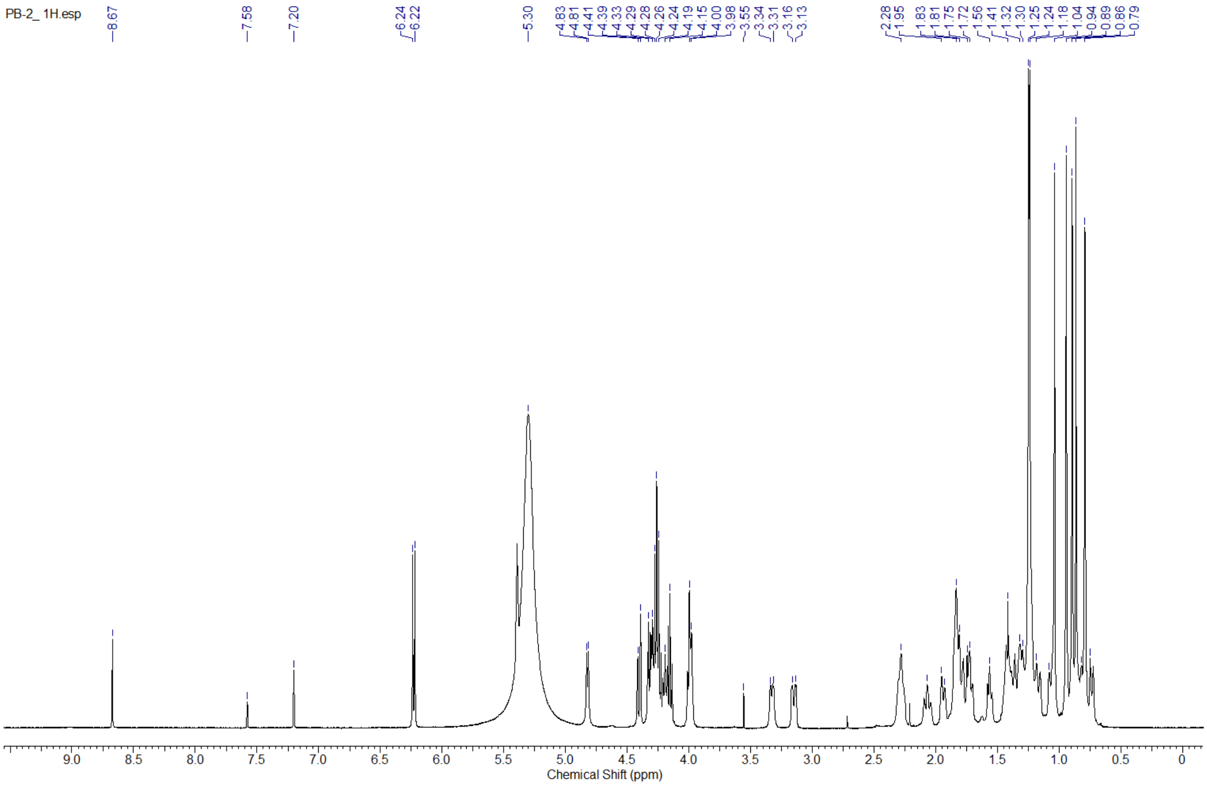

Figure S4. ^{1}H (500 MHz) spectrum of compound 2.

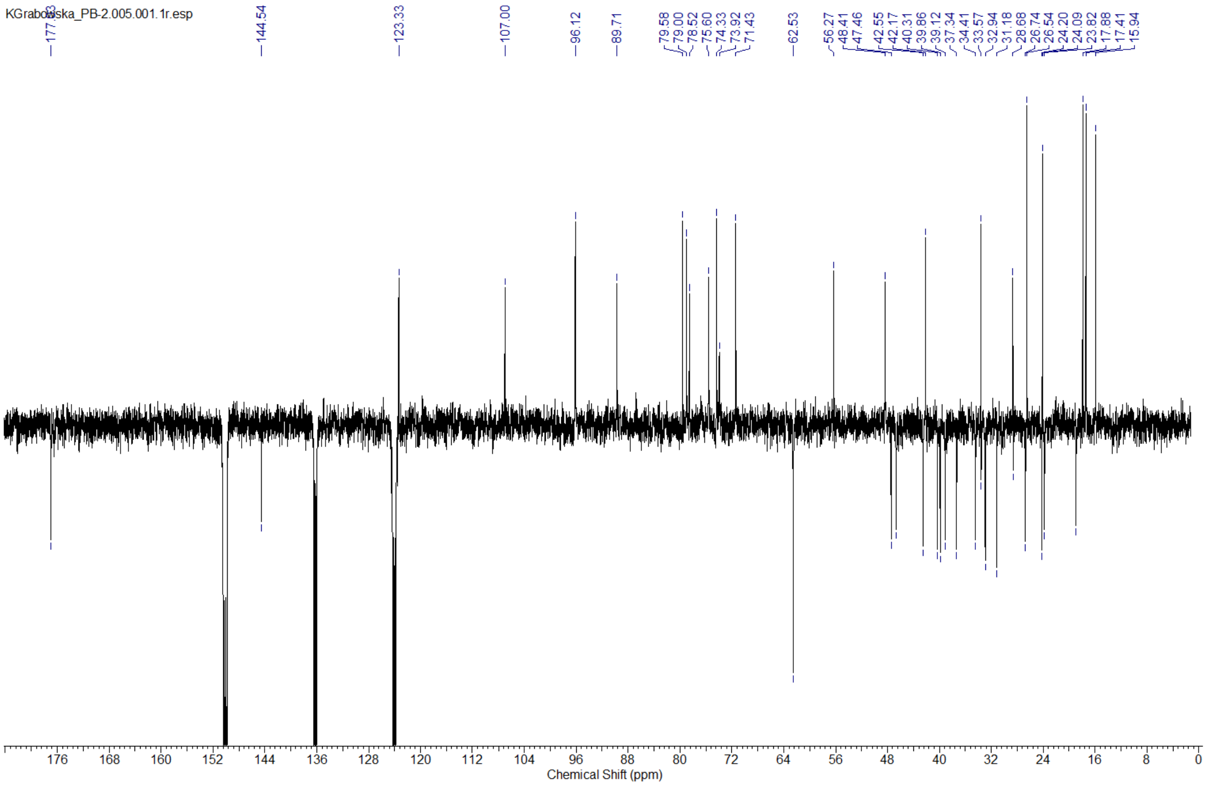

Figure S5. ^{13}C DEPT Q (125 MHz) spectrum of compound 2.

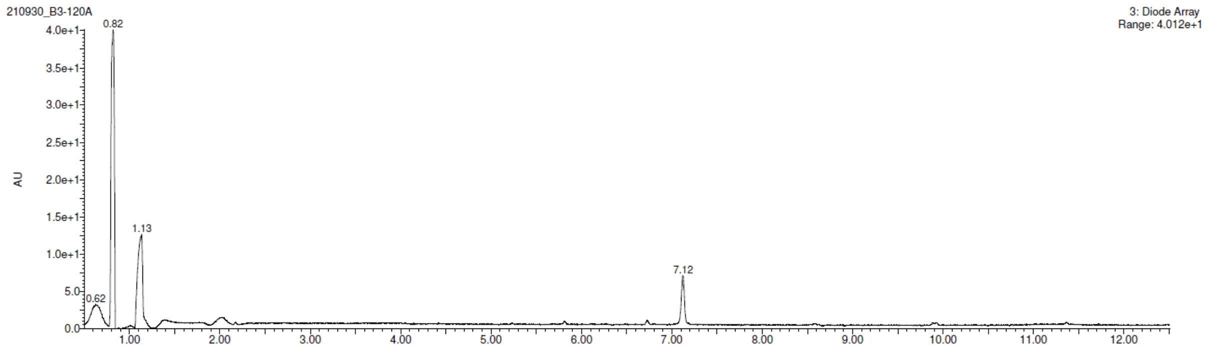

Figure S6. UPLC (PDA; range λ = 200-700 nm) chromatogram of compound 1

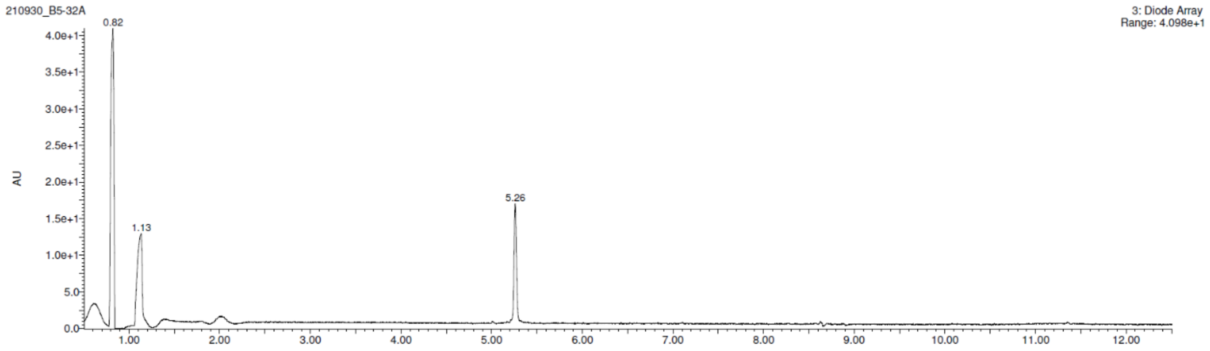

Figure S7. UPLC (PDA; range λ = 200-700 nm) chromatogram of compound 2.

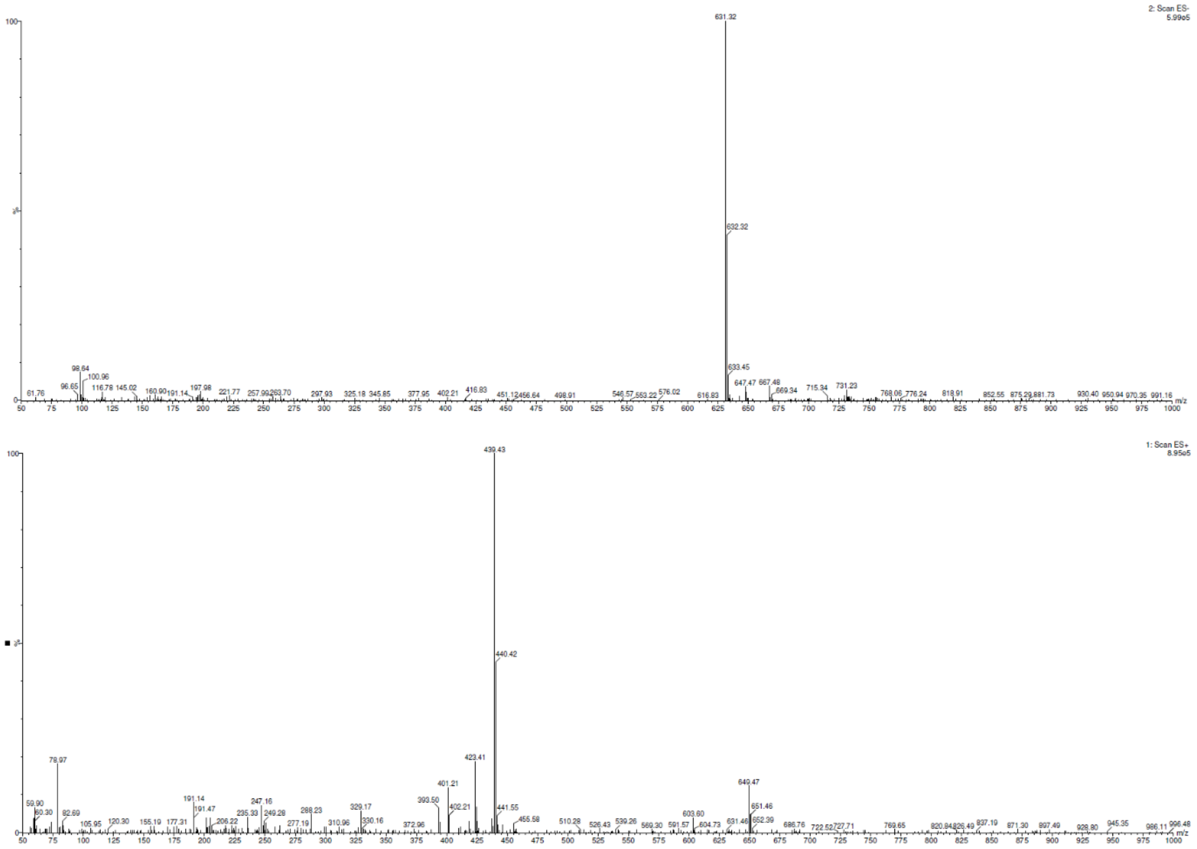

Figure S8. ESI-TQD-MS and MS/MS spectra (negative and positive ion mode) of compound 1.

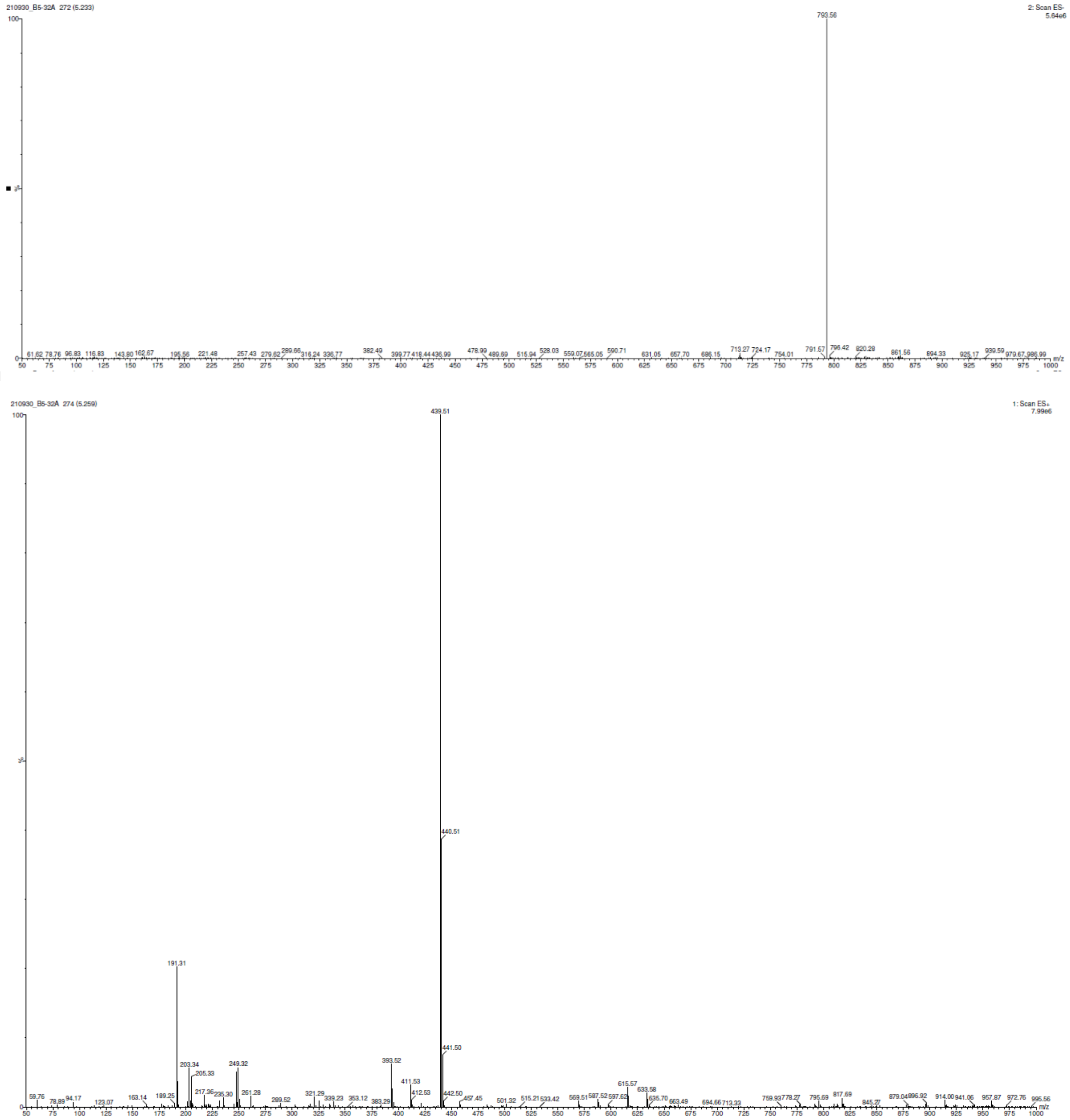

Figure S9. ESI-TQD-MS and MS/MS spectra (negative and positive ion mode) of compound 2.

Supplementary note 1: Structure elucidation

The negative-ion electrospray ionisation mass spectrometry (ESI-MS) of compound **1** exhibited a pseudomolecular ion peak at *m/z* 631.19 $[M - H]^-$ (deprotonated molecule), whereas the ESI-MS spectrum in positive ion mode displayed ion peaks at *m/z* 649.47, which were assigned to ammonium adduct $[M + NH_4]^+$ and diagnostic ion at *m/z* 439.43, corresponding to loss of GlcA residue and loss of water from the sapogenin $[M + H - 176 - 18]^+$. Furthermore, GlcA was identified as a constituent of the structure through acid hydrolysis of the compound. The analysis of ^{1}H NMR, DEPT-Q, and 2D NMR (HSQC, HMBC, H2BC, COSY, TOCSY) spectra enabled the identification of the aglycone as oleanolic acid. The downfield shift of C-3 (δ 89.70) indicated the presence of a glycosylation at this position. The linkage position of the glucuronic acid to the sapogenin was confirmed based on the long-range heteronuclear multiple bond correlation (HMBC) between the anomeric proton at δ 4.85 and the carbon signal at δ 89.70, which corresponds to the C-3 of the aglycone. The data obtained, when compared with existing literature [DOI: 10.1021/acs.jafc.0c04603 ; DOI: 10.1016/s0367-326x(99)00166-5 ; DOI: 10.1007/s10600-012-0216-2; DOI: 10.3390/molecules28030982], indicated that compound **1** is identical with oleanolic acid-3-*O*-β-D-glucuronopyranoside (calenduloside E).

In negative electrospray ionisation mass spectrometry, compound **2** exhibited a pseudomolecular ion peak at *m/z* 793.56 $[M - H]^-$. A comparison of the MS data for compounds **2** and **1** revealed a difference of 162 mass units, indicating that compound **2** has an additional hexose unit. In positive ion mode, the ESI-MS spectrum yielded an intense ion at *m/z* 439.51, resulting from the loss of a sugar moiety (comprising one hexose unit and a GlcA residue) accompanied by the dehydration of the sapogenin $[M + H - 162 - 176 - 18]^+$. The NMR spectrum of compound **2** also exhibited signals corresponding to two anomeric protons at δ 4.82 and δ 6.23. The results of the acidic analysis of compound **2** demonstrated the presence of GlcA and glucose in its structure. A comparison of the 1D and 2D NMR spectral data of the isolated compounds **1** and **2** revealed a high degree of similarity, with the exception of the signals corresponding to glucose, which were observed exclusively in the spectrum of compound **2**. Moreover, the downfield shift values of C-28 (δ 177.03) suggest that compound **2** has a 3,28-bidesmosidic structure. This was corroborated by the correlation between the anomeric proton of glucose (δ 6.23) and C-28 (δ 177.03 in the HMBC spectrum). In light of the aforementioned data and their comparison with existing literature [DOI: 10.1016/s0367-326x(99)00166-5 ; DOI: 10.3390/molecules28030982], it was determined that compound **2** is 3-*O*-β-D-glucuronopyranosyl oleanolic acid 28-*O*-β-D-glucopyranosyl ester, also known as chikusetsusaponin IVa.

Table S2. The basic features of PCA models constructed for compounds 1-4.

Model	Variance explained by the first two components (%)	Eigenvalues of first two principal components	Parameters mainly loaded on the first PC (with respective loadings)	Parameters mainly loaded on the second PC (with respective loadings)
CE	97.4	8.97; 0.77	conc. 4 (0.320)<br>conc. 6 (0.326)<br>conc. 10 (0.327)<br>conc. 20 (0.325)	conc. 0.5 (0.432)<br>conc. 1 (0.359)<br>conc. 50 (-0.408)<br>conc. 100 (-0.442)
ChIVa	98.4	8.90; 0.94	conc. 4 (0.329)<br>conc. 6 (0.332)<br>conc. 10 (0.334)<br>conc. 20 (0.330)	conc. 0.5 (-0.449)<br>conc. 1 (-0.363)<br>conc. 50 (0.402)<br>conc. 100 (0.541)
MIc	93.7	8.43; 0.94	conc. 6 (0.333)<br>conc. 10 (0.329)<br>conc. 20 (0.333)<br>conc. 30 (0.329)	conc. 0.5 (0.439)<br>conc. 1 (0.384)<br>conc. 50 (-0.437)<br>conc. 100 (-0.463)
OA	98.1	8.73; 1.08	conc. 6 (0.334)<br>conc. 10 (0.335)<br>conc. 20 (0.330)	conc. 0.5 (-0.389)<br>conc. 50 (0.462)<br>conc. 100 (0.550)

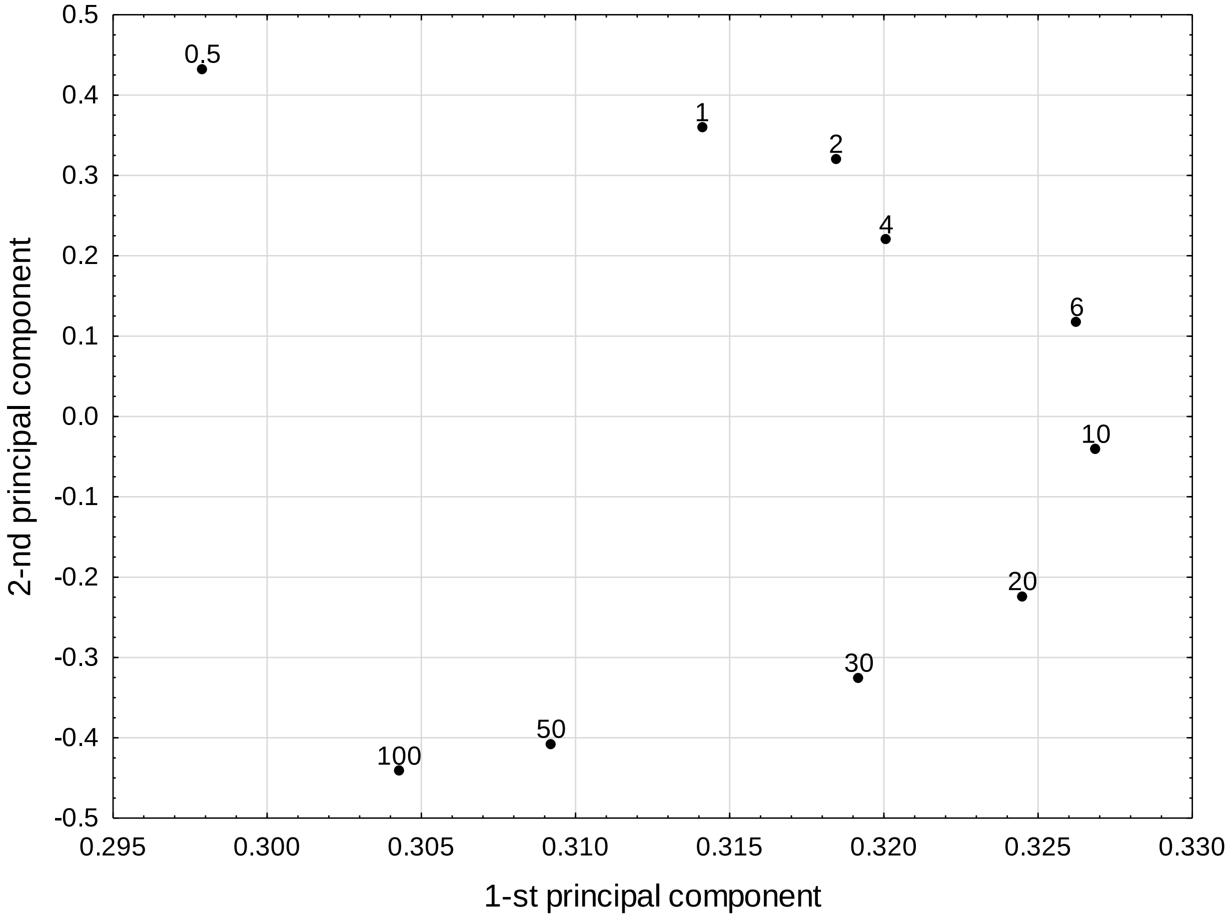

Figure S10. The loading scatter plot for model 1 (the numbers next to the points indicate the concentration of compound 1 in subsequent solutions, μg/mL).

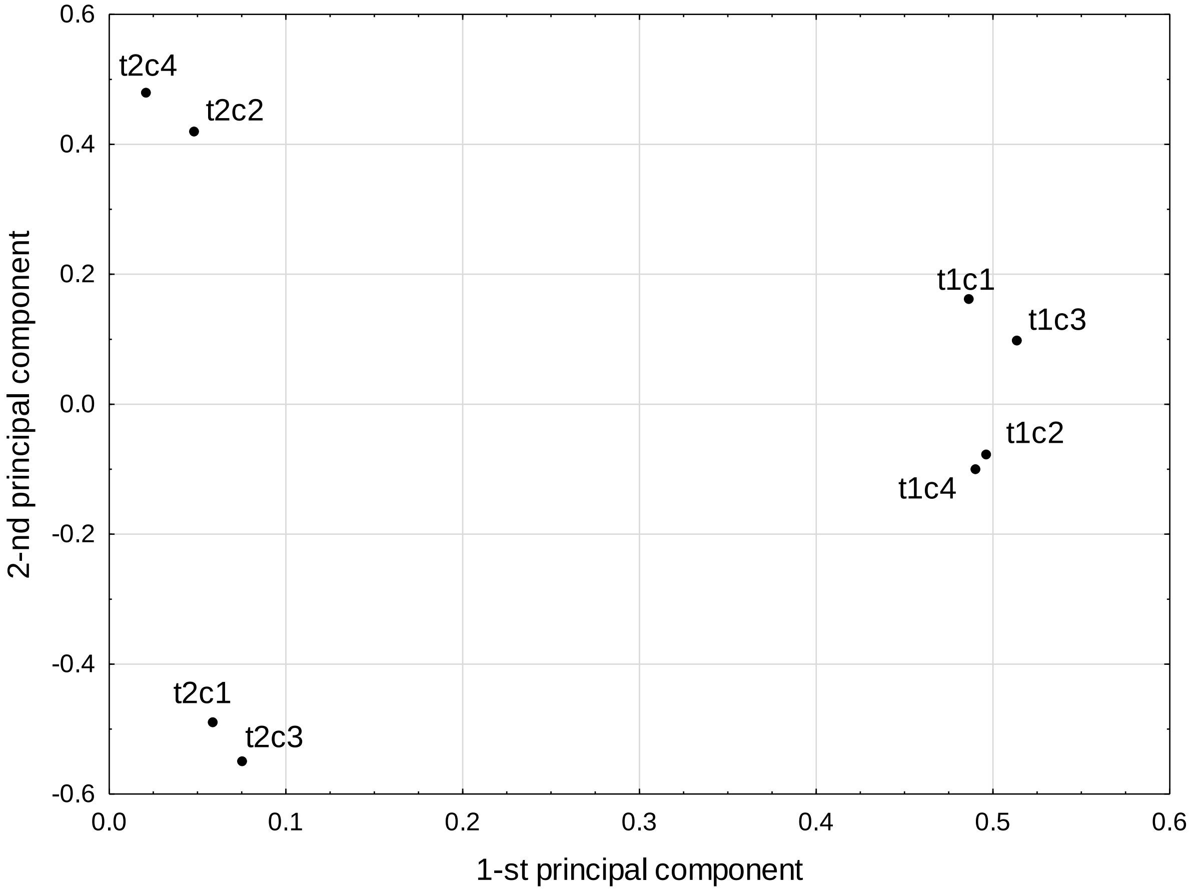

Figure S11. The loading scatter plot for hierarchical principal component analyses model (the symbol t1c1 denotes first principal component in PCA model for CE; the symbol t1c2 denotes second principal component in PCA model for CE; etc.).
